# A multiple phenotype imputation method for genetic diversity and core collection in Taiwanese vegetable soybean

**DOI:** 10.3389/fpls.2022.948349

**Published:** 2022-09-02

**Authors:** Yen-Hsiang Huang, Hsin-Mei Ku, Chong-An Wang, Ling-Yu Chen, Shan-Syue He, Shu Chen, Po-Chun Liao, Pin-Yuan Juan, Chung-Feng Kao

**Affiliations:** ^1^Department of Agronomy, College of Agriculture and Natural Resources, National Chung Hsing University, Taichung, Taiwan; ^2^Department of Agronomy, College of Bioresources and Agriculture, National Taiwan University, Taipei, Taiwan; ^3^Plant Germplasm Division, Taiwan Agricultural Research Institute, Taichung, Taiwan; ^4^Advanced Plant Biotechnology Center, National Chung Hsing University, Taichung, Taiwan

**Keywords:** vegetable soybean, germplasm, phenotypic diversity, core collection, edamame, multiple imputation, phenotypes, correlated samples

## Abstract

Establishment of vegetable soybean (edamame) [*Glycine max* (L.) Merr.] germplasms has been highly valued in Asia and the United States owing to the increasing market demand for edamame. The idea of core collection (CC) is to shorten the breeding program so as to improve the availability of germplasm resources. However, multidimensional phenotypes typically are highly correlated and have different levels of missing rate, often failing to capture the underlying pattern of germplasms and select CC precisely. These are commonly observed on correlated samples. To overcome such scenario, we introduced the “multiple imputation” (MI) method to iteratively impute missing phenotypes for 46 morphological traits and jointly analyzed high-dimensional imputed missing phenotypes (EC_*impu*_) to explore population structure and relatedness among 200 Taiwanese vegetable soybean accessions. An advanced maximization strategy with a heuristic algorithm and PowerCore was used to evaluate the morphological diversity among the EC_*impu*_. In total, 36 accessions (denoted as CC_*impu*_) were efficiently selected representing high diversity and the entire coverage of the EC_*impu*_. Only 4 (8.7%) traits showed slightly significant differences between the CC_*impu*_ and EC_*impu*_. Compared to the EC_*impu*_, 96% traits retained all characteristics or had a slight diversity loss in the CC_*impu*_. The CC_*impu*_ exhibited a small percentage of significant mean difference (4.51%), and large coincidence rate (98.1%), variable rate (138.76%), and coverage (close to 100%), indicating the representativeness of the EC_*impu*_. We noted that the CC_*impu*_ outperformed the CC_*raw*_ in evaluation properties, suggesting that the multiple phenotype imputation method has the potential to deal with missing phenotypes in correlated samples efficiently and reliably without re-phenotyping accessions. Our results illustrated a significant role of imputed missing phenotypes in support of the MI-based framework for plant-breeding programs.

## Introduction

Vegetable soybeans are soybeans [*Glycine max* (L.) Merr.] harvested in the R6 stage when pods and seeds are full but still green. The features of vegetable soybeans are large-seeded and high in nutrients. Although classified as legume crops, they are also regarded as vegetables with a low-input and a short life cycle (Zhang et al., 2017). Currently, vegetable soybean varieties are mainly from Japan and Taiwan for the world’s commercial production (Han and Gai, 2002). In the Japanese market, vegetable soybeans are usually sold with stalks and pods. Therefore, vegetable soybeans are also known as edamame, which means branched bean. They are rich in proteins, free amino acids, carbohydrates, vitamins, minerals, phytoestrogens, and edible oil (Hu and Lin, 2018). Compared to most crops, the fresh seeds of vegetable soybeans have a relatively higher protein content (Rao et al., 2002). The soybean protein is seen as a complete protein because of essential amino acids (Velásquez and Bhathena, 2007). Therefore, vegetable soybeans have found its way into the domestic and international market chains because of edamame’s nutritional properties and the trend for a healthier lifestyle (Ebert et al., 2017).

The soybean germplasm in different countries encompasses unique characteristics. For instance, Japanese populations differ from Chinese germplasm pools, whereas Korean accessions were involved in both (Abe et al., 2003). On the other hand, accessions from southeast and south/central Asia have relatively high genetic diversity (Abe et al., 2003). Therefore, the genetic diversity of worldwide varieties can bring plentiful germplasm resources to vegetable soybean breeding (Kaga et al., 2012). In Taiwan, the National Plant Genetic Resources Center (NPGRC) has collected and preserved abundant domestic landraces and germplasm accessions of vegetable soybeans from abroad. These collections came from the Taiwan Agricultural Research Institute, the Asian Vegetable Research and Development Center (AVRDC), National Chung-Hsing University, National Chiayi University, and many agricultural research institutes in Taiwan. Many Taiwanese vegetable soybean accessions (e.g., Ryokkoh, Tzurunoko, and Jikkoku) mainly originated from Japan, which has diverse gene pools from Chinese and United States edamame collections (Cui et al., 2000; Zhou et al., 2000), indicating that distinct genetic bases had been preserved in Taiwanese edamame accessions. For instance, the variety Ryokkoh has a brighter green pod color and has larger seed size and better flavor than the variety Tzurunoko (Shanmugasundaram et al., 1991). The variety Jikkoku was called “Shih Shih” in Taiwan and used as a multipurpose application in vegetable soybean variety improvement (Shanmugasundaram, 1976). Several varieties (Y-386, Vesoy #4, PI157424, Houjaku, Ryokkoh, Yoshida-1, Disoy, BPI #4, and Tzurunoko) are characterized by large seeds that were introduced from China, South Korea, the United States, and the Philippines. Improved varieties, such as Kaohsiung No. 2, No. 3, and Kaorihime, were characterized by heavier 100-immature seed weight, higher shelling rate, full of fresh pods, or special aroma (e.g., taro-flavor) (Arikit et al., 2011; Chou, 2016). The Taiwanese edamame collection preserved Taiwanese ancestors and exotic ancestors, which formed a rich and diverse genetic diversity and had a wide range of phenotypic traits. Hence, Taiwan has the largest and richest resources of abundant accessions with diverse genetic materials for phenotypic diversity, which provides opportunities to improve the breeding of targeted traits.

Vegetable soybean is mainly planted in Japan, Taiwan, China, Thailand, and Vietnam (Yinbo et al., 1997), and it is currently an important worldwide cash crop. However, collecting and preserving vegetable soybean accessions is difficult because of specific planting requirements and trade-off between grain soybean and vegetable soybean (Kao et al., 2021). Furthermore, phenotypic investigation and data collection of vegetable soybean germplasms have become more challenging because of limitations on experiment, labor cost, and environmental conditions. These reasons all caused difficulties in the collection of vegetable soybean seeds. This is why the core collection (CC) of vegetable soybean germplasm is less studied. The first CC of vegetable soybean was developed by our laboratory (Kao et al., 2021). A modified Roger’s distance algorithm was proposed to select 30 accessions (i.e., CC) for Taiwanese vegetable soybeans. In addition, we established a CC containing 23 accessions to be representative of five important traits (large seeds, stay-green pods, high isoflavone content, cold tolerance, and high yield) of vegetable soybeans (Chu et al., 2019).

In recent decades, many crop germplasms have been established. However, it is particularly difficult for biologists and breeders to efficiently obtain knowledge and information from enormous amounts of germplasm materials. Hence, the idea of CC for germplasms was first proposed by Frankel (1984). He defined CC as the minimum set of accessions representing maximum diversity with least redundancy of accessions in the entire collection (EC). This concept has been applied widely to construct a CC from a maximum collection of germplasms for plants, vegetables, and fruits (Pino Del Carpio et al., 2011; Oliveira et al., 2014; Yun et al., 2015) and capture the maximum genetic variation of accessions in a germplasm collection. Diversity investigation and CC establishment for soybean germplasms have been studied by many countries, including Canada (Fu et al., 2007), Japan (Kaga et al., 2012), South Korea (Jo et al., 2021), China (Li et al., 2008), the United States (Aldrich-Wolfe et al., 2015), and Indonesia (Sulistyo et al., 2019). However, some of the studies mentioned above (Li et al., 2008; Kaga et al., 2012) directly removed loci (single nucleotide polymorphisms and simple sequence repeats) because of missing genotypes, or discarded accessions because of missing phenotypes. Deletion methods (including listwise deletion and pairwise deletion) may result in biased results, which lead to the CC not being representative of the population. In view of this, a robust imputation approach is required to provide valid results and avoid loss of precision and power resulting from incomplete data.

With the development of sequencing technology, the efficiency of building a CC has been improved on account of acquisition of molecular markers to screen accession genotypes directly and combination of phenotypic data (Khaled and Hamam, 2015; Zhang et al., 2017). Studies pointed out that the use of combined phenotypic and genotypic data will have the best outcome and that using either one of them alone would yield less favorable results (Kumar et al., 2016). The effect depends on the quality control of the data (Lee and Simpson, 2014). Most importantly, a precise and accurate dataset is the primary key to obtaining representative core accessions for germplasms regardless of phenotype or genotype (Yun et al., 2015; Kao et al., 2021).

Studies on germplasm are the basis of crop breeding and improvement. However, the existence of missing values in phenotypes would severely affect the population structure and grouping of germplasms, and limits the investigation of germplasm diversity and establishment of CCs in germplasm accessions. Missing values have three forms, namely, missing completely at random (MCAR), missing at random (MAR), and missing not at random (MNAR). As seen in most germplasms, the phenotypes in our vegetable soybean germplasms also suffered from missingness because of cultivation problems in field trials and natural causes. Missing values mostly are unobserved or unrecorded data, which can be classified as MAR. In order to deal with missing data, many researchers use deletion methods such as listwise deletion and pairwise deletion or single imputation. Although these approaches are easily implemented, they may bias the result of diversity in interpreting germplasms (Taugourdeau et al., 2014; Poyatos et al., 2016). Incomplete phenotypic data and reduced level of genetic diversity would limit breeding progress (Wang et al., 2017).

Often accessions are missing or difficult in determining phenotypic traits limiting the diversity in collection and application of CCs. Accessions with missing phenotypes will under- or overestimate diversity, which is often complicated by missingness (Newman and Sin, 2009). The best way is to re-phenotype accessions. However, it is typically costly, infeasible, and time-consuming. Instead, multiple imputation (MI) is an algorithm for efficiently dealing with missing phenotype accessions. Several predictive datasets are created, and the estimates obtained from each imputed dataset are pooled (Sterne et al., 2009; Lee and Simpson, 2014). MI procedures can account for different sources of uncertainty computationally that arise from the imputation approach itself, model parameters, and residuals. If MAR is presented in data, MI will enable all accessions to be included in the analysis and provide valid results (Royston, 2004; Lee and Simpson, 2014). Also, the MI method is a better approach for missing data that are a mixture of MAR and MNAR. We noted that the reliability and accuracy of imputed values are inversely proportional to the missing rate. Taken together, MI has the potential to improve statistical validity in agricultural research, and is an efficient alternative solution for incomplete phenotypes.

To explore the population structure of vegetable soybean germplasms, a cluster analysis is required to reveal the distribution of germplasm accessions and determine whether the CC captures the most diversity richness and evenness of the EC. Weighted *k*-means clustering is an unsupervised algorithm to iteratively search for the solution to clustering multiple correlated phenotypes across multiple correlated accessions (Foss and Markatou, 2018). Also, it is able to handle mixed-type quantitative and qualitative traits simultaneously. With unsupervised learning algorithms, pairwise similarities (or dissimilarity) across accessions can be determined to ascertain an appropriate number of clusters having a good partition among clusters.

Many indices of summary statistics and statistical criteria were developed to characterize genetic diversity and to evaluate CC quality in a species. These indices use frequency-based data to numerically describe diversity in terms of number of different traits (i.e., richness) and relative abundance of traits (i.e., evenness) present in a particular species. Five diversity indices were commonly used for species diversity investigation, namely, the Shannon–Weaver, Nei’s diversity, Simpson’s diversity, Margalef, and Pielou’s indices. Among them, the Shannon–Weaver diversity index was the best index to assess the richness and diversity of a species (Kumar et al., 2022). Nei’s diversity index was used widely in studies on the literature as a criterion for evaluating phenotypic or genetic diversity of the CC in a species (Zhang et al., 2012; Odong et al., 2013; Schafleitner et al., 2015; Mahmoodi et al., 2021). In addition, Jain et al. (1975) and Yan et al. (2010) demonstrated the effectiveness of both diversity indices in exploring germplasm resources and geographical patterns. Hence, monitoring of plant species diversity, evenness, and richness are essential for a better understanding of diversity patterns and complex phenological phenomena for plant breeding.

Incomplete phenotypic information directly affects the results of CCs and low resource usage efficiency of germplasms. Since there is a varying degree of missing values in the vegetable soybean phenotypic trait data, it could impact the result of germplasm cluster analysis, and CC construction could be impacted. In order to minimize the influence, this study mainly focused on dealing with missing values in phenotypic traits (i.e., incomplete entire collection, EC_*raw*_), and we created a complete (i.e., observed plus imputed values) collection (EC_*impu*_). Then, we used the advanced M strategy algorithm to construct a CC (including the CC_*raw*_ and CC_*impu*_) with the PowerCore software. Furthermore, we conducted difference tests, clustering analyses, and diversity investigation for both each and overall individual traits to evaluate the representativeness of the CC. The five assessment indices for the CC were used to evaluate the representativeness of the core set of Taiwan vegetable soybean germplasm. Finally, the impact on input data (i.e., number of phenotypic traits) and number of core collection accessions under a range of thresholds of missing phenotype rates was addressed and discussed. The framework applied in this study suggests a solution to figure out the difficulty arising from missingness efficiently and provides an opportunity for assessing the genetic architecture of complex phenotypes among correlated accessions.

## Materials and methods

### Vegetable soybean germplasm

A total of 213 vegetable soybean germplasm accessions and 47 phenotypic traits collected and preserved from the NPGRC were utilized in this study. The origins of the vegetable soybean accessions were Taiwan, Japan, China, Hong Kong, South Korea, the United States, the Philippines and unknown origins. Phenotypic traits were investigated and recorded in the field at Kaohsiung District Agricultural Research and Extension Station, COA, complied with the guidelines of distinctness, uniformity and stability test, in four consecutive autumn cropping seasons (1995–1998). For more details, please refer to Kao et al. (2021).

The phenotypic traits (21 quantitative and 26 qualitative phenotypes) were characterized by a wide range of features regarding 38 morphologic phenotypes, 5 growth phenotypes, 2 phenological phenotypes, and 2 production phenotypes. The 38 morphologic phenotypes include seed length (mm), seed width (mm), seed thickness (mm), leaflet length (cm), leaflet width (cm), pod length (cm), pod width (cm), single pod weight (g), number of pods per 500 g, number of seeds per pod, shelling rate (%), immature seed length (mm), immature seed width (mm), immature seed thickness (mm), seed shape, seed color, hilum color, hypocotyl coloration, number of nodes on main stem, stem color, number of branches, leaflet size, leaflet shape, number of leaflets, leaf color, pubescence density, pubescence color, corolla color, pod set capacity, pod length, pod width, pod shape, pod color, immature seed size, immature seed coat color, immature seed texture, easiness of pod removal, and storability. The five growth phenotypes include internode length (cm), plant height (cm), first pod height (cm), lodging score, and plant type. The two phenological phenotypes are from sowing to flowering (days) and from blooming to harvest (days). The two production phenotypes are 100 seed weight (g) and 100 immature seed weight (g). All the phenotypic traits were recorded by observation of at least 10 randomly selected plants from each replication in a randomized complete block design with four replicates. We noticed that five phenotypic traits (number of nodes on main stem, number of branches, lodging score, pod length, and pod width) were categorized into qualitative traits. The criteria are described below. The number of nodes on main stem was classified into small (less than 13 nodes), medium (between 13 and 17 nodes), or large (more than 17 nodes). The number of branches was partitioned into low (less than 4 branches), medium (between 4 and 5 branches), or high (more than 5 branches). Lodging score was calculated from the leaning angle that can be grouped into absent (lower than or equal to 10°), medium (between 20 and 40°), or high (greater than 40°). Pod length was classified into short (less than or equal to 4.4 cm), medium (between 4.4 and 5 cm), or long (greater than or equal to 5 cm). Pod width was grouped into narrow (less than or equal to 1.1 cm), medium (between 1.1 and 1.3 cm), or broad (greater than 1.3 cm).

### Meteorology data

The meteorology data were obtained from two institutes of the Kaohsiung District Agricultural Research and Extension Station (KDARES) and the Kaohsiung Weather Station of the Central Weather Bureau in Taiwan. The records included daily data during 1995–1998 on temperature (°C), humidity (%), sunshine duration (hours), precipitation (mm), and days with precipitation (day). We previously examined and revealed that the phenotypic data recorded by experienced experts who were well-trained on phenotypic investigation in the fields at KDARES had no significant environmental effects. For detailed results, please refer to Kao et al. (2021).

### Multiple phenotype imputation of missing phenotypes

Multiple phenotype imputation is a solution of providing a valuable imputed value for handling missing data in multiple correlated phenotypes observed on correlated samples (Dahl et al., 2016). Three major steps were applied to handle missing phenotypes: imputation, estimation, and pooling of estimates (Papageorgiou et al., 2018). First, unobserved phenotypes were repeatedly generated to capture the sources of uncertainties during the MI procedures. Applying Bayesian model regression, the imputed values were randomly sampled from the predictive distribution based on observed data (Lee and Simpson, 2014). For each accession with unobserved data, we fit the model


$$Y_{i}^{*}∼N\left(u_{i},\sigma_{i}^{2}|Y_{\backslash Y^{*}}\right)$$

to estimate missing data for a specific phenotypic trait; $Y_{i}^{*}$ is the partial unobserved phenotype in the *i^th^* trait, and *Y*_\Y*_ is the maximum dataset of the observed phenotype. Thus, the posterior mean (*u_i_*) of the multivariate normal distribution can be used to impute missing phenotypes (Y*). In the estimation stage, the estimated associations in each imputed dataset would differ because of the variability of imputed values, so the outcomes differed slightly among each imputation. This is because all imputed phenotypes will not be distributed on the regression line so the data’s true variability can be obtained (Kleinke and Reinecke, 2015). The imputed values of qualitative traits were estimated from posterior predictive distribution, while those of quantitative traits were estimated from predictive mean matching (Gelman and Hill, 2011; Kropko et al., 2017). As for the pooling stage, the estimated results of the multiple imputation were pooled by taking the mean of the estimates. The $\hat{\text{R}}$ statistic was computed to verify the iterative convergence of the MI procedures. To meet the criterion of convergence, a $\hat{\text{R}}$ statistic less than 1.1 is required (Gelman and Hill, 2011; Su et al., 2011). To better generate the imputed phenotypes for missingness, we conducted thirty iterations in the first stage, followed by five iterations in the second stage with chained equations (four independent chains) during the MI process. The *mi* package in R was used in the analysis.

### Measurement of correlation

Correlations were calculated between traits and visualized in a correlation matrix heatmap. We used Pearson’s correlation coefficient to measure the statistical relationship (i.e., strength and direction of association) between quantitative traits. Spearman’s correlation coefficient was used to measure the association between qualitative traits. We classified the quantitative traits into categories according to the result of the clustering analysis and applied Spearman’s correlation to calculate the correlation between a qualitative trait and a categorized quantitative trait. Point-biserial correlation coefficient was used to measure the association between a dichotomous trait (nominal trait with only two levels) and a quantitative trait (Khamis, 2008). The value of correlation coefficient lies between –1 (perfect positive) and +1 (perfect negative). Germplasm accessions almost always use related accessions. Therefore, the MI method is beneficial to the estimates of missing phenotypes. The MI procedures can potentially boost power to uncover population structure and relatedness kinship among high correlated phenotypes across correlated accessions (Dahl et al., 2016).

### Construction of core collection

We established the core collection on PowerCore software version 1.0. PowerCore is a widely used software package for establishing CCs. This program uses an advanced M (maximization) strategy with a modified heuristic algorithm (Kim et al., 2007). The M strategy has been used to select representative accessions with maximum coverage to attain a limited core set of the EC depending on the level of variability in germplasms. Quantitative phenotypic traits were classified into different classes based on the Sturges’ rule (Sturges, 1926), which is defined as *K* = 1 + *log*_2_(*n*), where *K* is the number of classes and *n* is the observed number of accessions. Qualitative phenotypic traits were grouped based on number of distinct characters. Finally, the modified heuristic algorithm was used to select the CC from the EC (EC_*raw*_ and EC_*impu*_) using 46 mixed-type phenotypic traits of vegetable soybeans so that the CC has minimum redundant accessions and maximum diversity.

### Weighted *k*-means clustering

We applied an unsupervised learning strategy through the weighted *k*-means clustering algorithm to search for the optimal number of cluster so that the genetic architecture feature of population structure and relatedness kinship of multiple mixed-type phenotypes among vegetable soybean germplasm accessions can be uncovered. For the first step in this approach, we set several *k* initial centers (i.e., two to fifteen, say), and based on the clusters, each germplasm accession was assigned to the neighboring centers using a dissimilarity measure. Weight was computed for each trait in each cluster and considered in the computation of dissimilarity measure (Badih et al., 2019). We set the weight as 0.5 by default. The optimal number of clusters (*k*) is characterized by high diversity and evenness (Shannon–Weaver diversity index >90%, Nei’s diversity index >80%), minimal intra-cluster distance, and maximal inter-cluster distance (variance explained >75%). Finally, to demonstrate the genetic diversity and structure of the germplasms, the *fpc* package in R (Hennig and Imports, 2015) was used for graphical representation of the results of the cluster analysis. Likewise, this algorithm was applied to individual quantitative traits to uncover the optimal number of clusters and reveal diversity. We applied the *wskm* and *kamila* packages in R to perform weighted *k*-means clustering (Foss and Markatou, 2018; Zhao et al., 2020).

### Phenotypic diversity analysis

Richness and uniformity are the two primary indices to evaluate phenotypic diversity. Richness represents the total number of clusters. Uniformity represents the degree of germplasm accessions in each of the clusters evenly distributed. When evaluating the property of germplasm, the larger the diversity index is, the higher the evenness of the phenotypic traits will be. The phenotypic diversity analysis was conducted using the Shannon–Weaver diversity index (Shannon and Weaver, 1949) and Nei’s diversity index (Nei, 1973). The Shannon–Weaver diversity index (*H’*) is defined as

• $\mathrm{H’}=-\sum_{i=1}^{S}\frac{p_{i}ln\left(p_{i}\right)}{\text{ln}\left(S\right)}$,

and Nei’s diversity index (*Nei*) is defined as

• Nei=1-∑i=1Spi2,

where *S* is the total number of clusters, *p*_*i*_ is the proportion of accessions in the *i^th^* cluster to the total number of germplasms. The value of *H’* and Nei is bounded between 0 and 1, and between 0 and (1-1/S), respectively. For estimating biodiversity richness and evenness, the Shannon–Weaver diversity index has more weight on genetic richness, and the Nei’s diversity index has more weight on genetic evenness (Kim et al., 2017).

### Evaluation of the core collection

A homogeneity test (Levene’s test) for variances and a difference test (*t*-test) for means were performed to determine the difference in phenotypic traits between the CC and the EC. Levene’s test was conducted by 1,000 times of bootstrap iterations to check the homogeneity of variances among the groups (Levene, 1960; Joseph et al., 2020). Then, an independent *t*-test was conducted to identify the significant difference in means between the CC and the EC. For quantitative traits, Student’s *t*-test was performed on those with equal variances, and Welch’s *t*-test was performed on those with unequal variances. For quantitative traits, Chi-squared test of homogeneity was performed to test the difference between the CC and the EC in the classification ratio of each trait (*p*-value <0.05).

We used five indices to evaluate whether the CC is representative and divergent from the germplasm or not, which included (1) the mean difference percentage ($\mathrm{MD}\%=\frac{1}{m}\sum_{i=1}^{m}\frac{\left|M_{e}-M_{c}\right|}{M_{c}}\times 100\%$), (2) variance difference percentage ($\mathrm{VD}\%=\frac{1}{m}\sum_{i=1}^{m}\frac{\left|V_{e}-V_{c}\right|}{V_{c}}\times 100\%$), (3) coincidence rate ($\mathrm{CR}\%=\frac{1}{m}\sum_{i=1}^{m}\frac{R_{c}}{R_{e}}\times 100\%$), (4) variable rate ($\mathrm{VR}\%=\frac{1}{m}\sum_{i=1}^{m}\frac{CV_{c}}{CV_{e}}\times 100\%$), and (5) coverage ($\mathrm{Coverage}\%=\frac{1}{m}\sum_{i=1}^{m}\frac{D_{c}}{D_{e}}\times 100\%$), where *M, V, R, CV, D* and *m* represent the mean, variance, range, coefficient of variation, number of clusters, and number of traits, respectively. As for the subscript, *e* is short for the EC while *c* is short for the CC. If *MD*% is lower than 20%, *VD*% is small enough; if *CR*% is greater than 80%, VR% is great enough, and if the coverage is close to 100, we assessed the CC to be well represented by the EC (Hu et al., 2000).

## Results

Initially, we had a total of 213 vegetable soybean germplasm accessions and 47 morphological traits available. After removing 13 redundant accessions (i.e., containing the same phenotypic data) and one trait (i.e., identical values in the “number of leaflets” trait had no meaning), 200 accessions and 46 phenotypic traits (25 qualitative traits and 21 quantitative traits) remained and were taken as the entire collection (denoted as EC_*raw*_).

**FIGURE 1:**
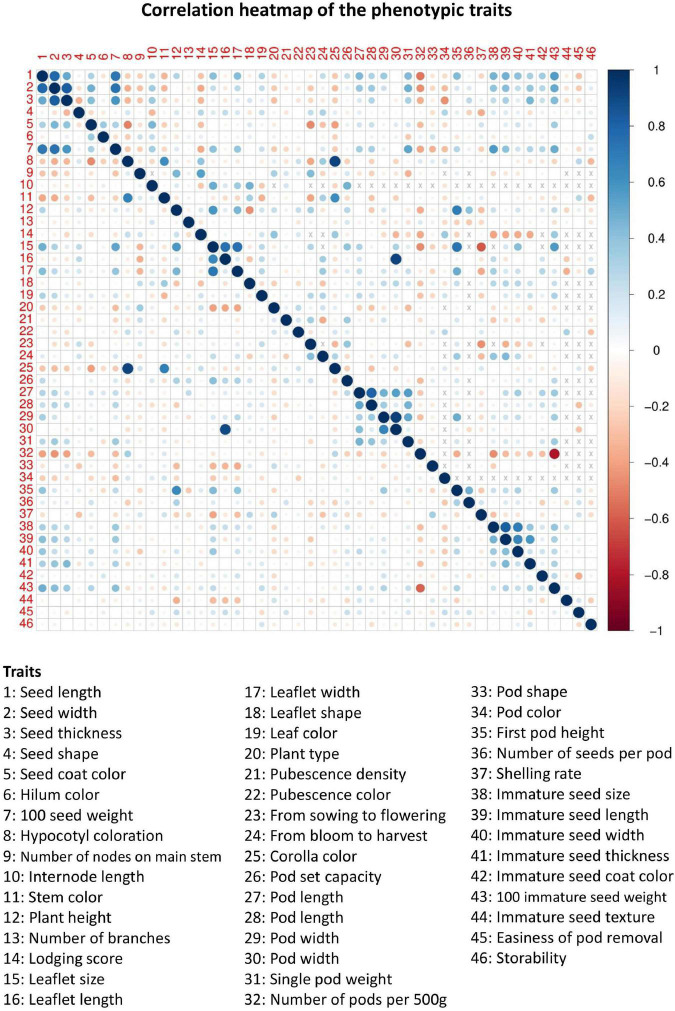
Correlation heatmap of 46 phenotypic traits in Taiwanese vegetable soybean accessions. The (top right panel) heatmap is based on observed phenotypes (EC_*raw*_), and the (bottom left panel) heatmap is based on complete (i.e., observed plus imputed values) phenotypes (EC_*impu*_). The size and color intensity of the circle are proportional to the degree of correlation. Positive and negative correlations are colored in blue and red, respectively. The symbol “×” means value is not available.

**FIGURE 2:**
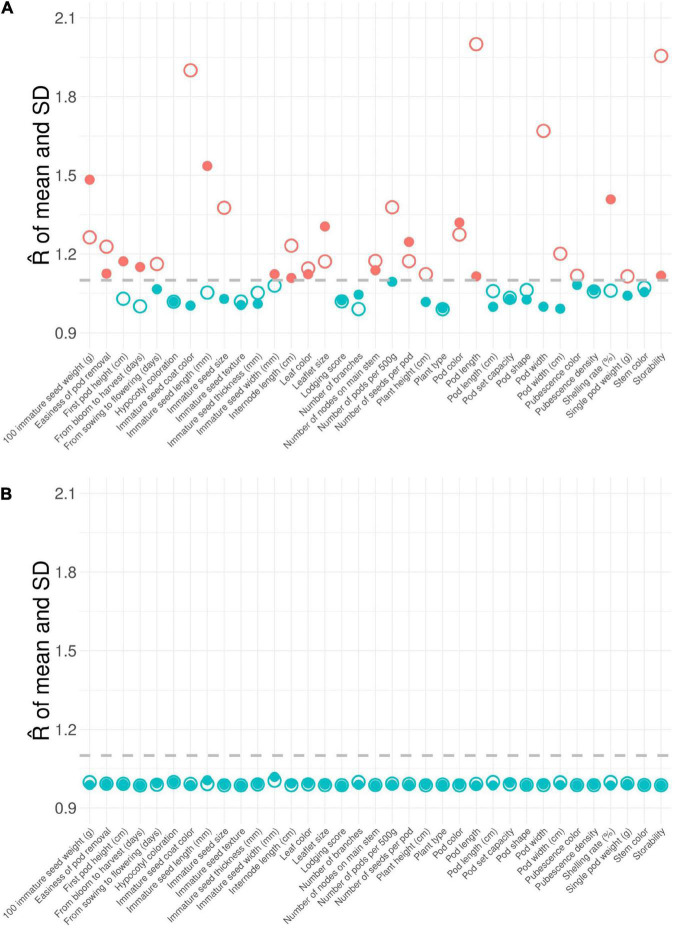
$\hat{\text{R}}$ statistic of imputed phenotypic traits with chained equation in multiple phenotype imputation. **(A)** First stage and **(B)** second stage of multiple phenotype imputation. The value of the $\hat{\text{R}}$ statistic is a convergence criterion for imputation. The imputation is converged (colored in blue) if $\hat{\text{R}}<$ 1.1 (or > 1.1) for all the imputed phenotypes but is non-converged otherwise (colored in red). The dashed line is defined as the convergent threshold of 1.1. The large hollow circle symbol represents the $\hat{\text{R}}$ statistic of mean. The small solid circle symbol represents the $\hat{\text{R}}$ statistic of standard deviation.

Of the 46 morphological traits, about 15.76 and 73.03% of phenotype pairs were strongly or moderately and weakly correlated among the 200 accessions, respectively (the top right panel of Figure 1 and Supplementary Table 1). Among them, approximately 33% of the phenotype pairs reached a statistically significant correlation (*p*-values less than 0.05), suggesting that multiple phenotypes are highly correlated. The correlations of the remaining phenotype pairs (11.21%) were unavailable because of unmatched phenotypes. In addition, different degrees of missingness that ranged between 0.5 and 78.5 percent occurred in forty (87%) phenotypes (Supplementary Tables 2, 3). Among the 46 phenotypic traits in our germplasms, 6 had no missing data, 23 had low missing rates (0.5–26.5%), 8 had moderate missing rates (49.5–52.5%), and 9 had high missing rates (64.5–78.5%) (Supplementary Tables 2, 3). By applying the chained equations in multiple phenotype imputation, all missing phenotypes across multiple correlated traits in related samples were filled by the imputed ones (denoted as EC_*impu*_). For each phenotypic trait, a $\hat{\text{R}}$ statistic was calculated to check convergence. The convergence criterion is defined as the $\hat{\text{R}}$ statistic in the second stage of the MI procedure that needs to be less than 1.1 by default, and is recommended by Gelman and Hill (2011). Obviously, more than half of the imputed phenotypes were not converged (i.e., $\hat{\text{R}}$ > 1.1) in the first stage of multiple phenotype imputation (Figure 2A). However, all imputed phenotypes were converged with $\hat{\text{R}}$ statistics less than the default threshold of 1.1 (Figure 2B) in the second stage, indicating stable and reliable estimates for missing phenotypes. For complete phenotypic traits, please refer to Supplementary Material 1. Twenty quantitative traits (except for the “from blooming to harvest” trait) demonstrated non-significant (i.e., *p*-values greater than 0.05) central tendency and dispersion of trait characteristics between complete (observed plus imputed values) and observed phenotypes (Supplementary Table 4). Likewise, nineteen qualitative traits (excluding “number of nodes on main stem,” “lodging score,” “plant type,” “pod shape,” “pod color,” and “immature seed texture”) showed non-significant (*p*-values greater than 0.05) frequency distribution of categorical traits between complete and observed phenotypes (Supplementary Table 5). Approximately 85% of complete phenotypes demonstrated consistent patterns of distributions with observed phenotypes, with only 15% having marginal difference, which are negligible for imputed data. As shown in the bottom left panel of Figure 1, a similar pattern of multiple correlated phenotypes was observed on complete phenotypes. The correlation coefficients of complete (observed plus imputed values) phenotype pairs ranged between –0.58 and 0.92. Noticeably, smaller circle sizes and lighter colors represented a marginal decline in the correlations among complete phenotypes, but a 10% increase was observed in significant phenotype pairs compared to observed phenotypes. Taken together, this suggests that our imputed phenotypes are reliable and representative.

**FIGURE 3:**
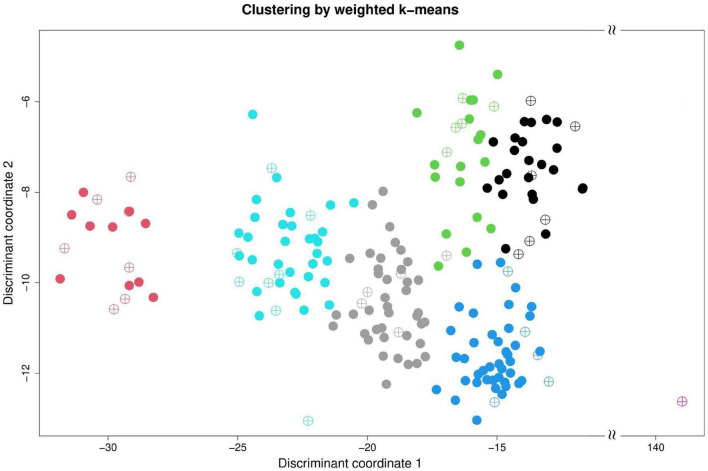
Cluster analysis of Taiwanese vegetable soybean accessions. Weighted *k*-means clustering was performed on 200 accessions using 46 complete (i.e., observed plus imputed values, EC_*impu*_) phenotypic traits. Taiwanese vegetable soybean germplasm accessions are classified into seven clusters. The solid (•) and N-ary circled plus operator (⊕) represents 200 accessions in the EC_*impu*_ and 36 selected accessions in the CC_*impu*_, respectively.

The clustering result of the Taiwanese vegetable soybean germplasm accessions can be classified into seven distinct clusters (Figure 3) according to genetic distance (or accessions similarity) across 46 mixed-type phenotypes using the weighted *k*-means clustering algorithm. All accessions of the EC_*impu*_ (•symbol) are distributed in all the seven clusters representing the 77.8% variance explained by clustering (Table 1). Accessions in the same cluster have close or similar characteristics of phenotypes compared to those outside the cluster. On the contrary, accessions between different clusters have diverse features across phenotypic traits. Among the EC, one accession named Sakata Kairyo Mikawashima (KG0192, a Japanese variety) may contain distinctive characteristics and specific genetic diversity that are very different from our germplasms; hence it was selected as one of the core accessions in the present study (i.e., both the CC_*impu*_ and the CC_*raw*_) and in our previous study (Kao et al., 2021). The seven clusters were characterized by large diversity richness and evenness (Shannon–Weaver diversity index = 0.9, Nei’s diversity index = 0.82) based on the familial relatedness and population structure of EC_*impu*_ explaining 77.8% of the genetic architecture of phenotypic features (Table 1). This indicates rich genetic variability of phenotypic features in the Taiwanese vegetable soybean germplasm accessions.

**TABLE 1:** Overall diversity and distribution of clustering of germplasm accessions in different collections (EC_*impu*_, CC_*impu*_, and CC_*raw*_).

Accessions collection	Distribution of clustering*^c^*							Variance explained	Overall diversity		
	1	2	3	4	5	6	7		H’	Nei’s	Retained/Lost
EC_*impu*_(200 accessions)	40	29	1	17	23	45	45	77.8%	0.90	0.82	
CC_*impu*_*^a^* (36 accessions)	8	6	1	8	4	5	4		0.94	0.83	Retained
CC_*raw*_*^b^* (43 accessions)	7	5	1	8	8	5	9		0.94	0.83	Retained

H’, Shannon–Weaver diversity index; Nei’s, Nei’s diversity index.

^a^The CC*_impu_* was selected with PowerCore using complete (observed plus imputed values) phenotypes (EC*_impu_*).

^b^The CC*_raw_* was selected with PowerCore and observed phenotypes (EC*_raw_*).

^c^Clustering analyses for mixed-type traits were conducted using the weighted *k*-means clustering algorithm.

In this study, we applied PowerCore to construct a sub-collection containing 36 accessions (CC_*impu*_) having minimum accessions and maximum diversity of the EC_*impu*_ (Table 2). The advanced M (maximization) strategy, through a heuristic algorithm, was applied to guarantee that the selected CC_*impu*_ had minimal redundancy and retained the maximum overall diversity of the whole germplasm accessions. The CC_*impu*_ (the ⊕ symbol in Figure 3) were distributed evenly across all the seven clusters, which cover the countries of origin Taiwan, Japan, South Korea, United States, Hong Kong, the Philippines, and unknown origins. A more even proportion of accessions in each cluster was found in the CC_*impu*_ (ranging from 2.78 to 22.22%) compared to that in the EC_*impu*_ (ranging from 0.5% to 22.5%). Furthermore, the overall diversity richness and evenness (Shannon–Weaver diversity index = 0.94, Nei’s diversity index = 0.83) of the whole germplasm accessions were retained efficiently in the CC_*impu*_, indicating that the selection of the core accessions was effective (Table 1). We noted that the same diversity richness and evenness were also retained in the CC using observed phenotypes (denoted as CC_*raw*_). Twenty-one accessions (∼60%) painted with a gray background represent the identical core accessions chosen by the CC_*raw*_ (Table 2), suggesting that the MI-based CC is efficient and reliable.

**TABLE 2:** Selected accessions in the core collection (CC_*impu*_) of Taiwanese vegetable soybean germplasms.

Germplasm ID*^a^*	Accession name*^b^*	Origin	Germplasm ID*^a^*	Accession name*^b^*	Origin
KG0001	ESB-66-3	Taiwan	**KG0125**	Chen Hsiang	Taiwan
KG0002	ESB-66-6	Taiwan	KG0128	Hsiao Ching	Unknown
**KG0009**	ESB-67-9	Taiwan	**KG0132**	Mainland China	Hong Kong
**KG0011**	ESB-67-14	Taiwan	KG0134	Taimeifood No.1	Taiwan
KG0015	Chakaori	Japan	KG0140	AGS188 (PI 187154)	Japan
KG0027	Erimo	Japan	KG0147	Hatsutaka	Japan
**KG0031**	G10493	Japan	KG0149	Ryuhyo	Japan
**KG0038**	G10502	Japan	**KG0153**	Kaohsiung No.2	Taiwan
**KG0050**	Fubaye	Unknown	KG0156	GC 83006-7	Taiwan
KG0054	Tamasudare	Japan	**KG0163**	D-62-7815	United States
KG0060	Tung Yeh	Japan	**KG0164**	Nuli-6-G2657	South Korea
KG0072	Gokuwase	Japan	KG0165	Nuli (PI 408251)	South Korea
KG0073	GokuwaseHayabusa	Japan	KG0167	Hua Yu 74-48	Taiwan
KG0086	Kaohsiung No.3	Taiwan	KG0170	KS1625	Taiwan
KG0088	Taisho Shiroge	Japan	**KG0180**	Yukinoshita-28	Japan
KG0092	PI 157424	South Korea	**KG0185**	AGS186 (PSB-VS 3)	Philippines
**KG0101**	Ryokukou	Japan	**KG0192**	Sakata KairyoMikowashima	Japan
**KG0106**	Kamui	Japan	**KG0196**	TzurarokoDaizu	Japan

^a^The germplasm ID highlighted in bold represents the identical accessions of CC based on 29 phenotypes reported by Kao et al. (2021); ID with gray background represents the identical accessions of the CC*_raw_*.

^b^The 36 accessions were selected as the core collection (CC*_impu_*) using the advanced M (maximization) strategy through a heuristic algorithm in the PowerCore software.

**FIGURE 4:**
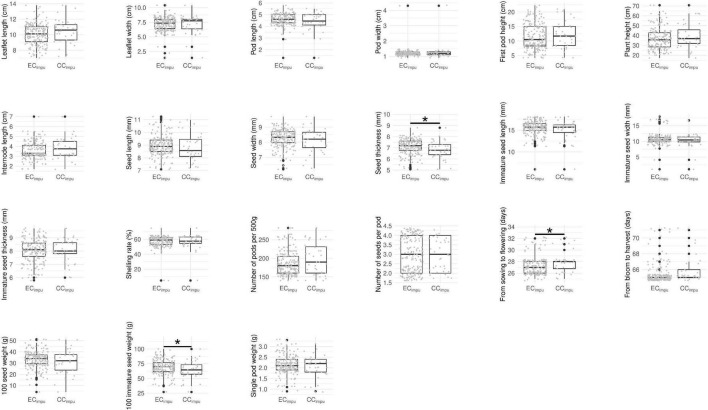
Boxplot of quantitative traits in the entire collection (i.e., observed plus imputed values, EC_*impu*_) and the core collection (CC_*impu*_). Gray dot represents individual accession. Asterisk represents significant difference between the CC_*impu*_ and the EC_*impu*_ by Student *t*-test (for equal variances) and Welch’s *t*-test (for unequal variances). *p-value < 0.05.

**FIGURE 5:**
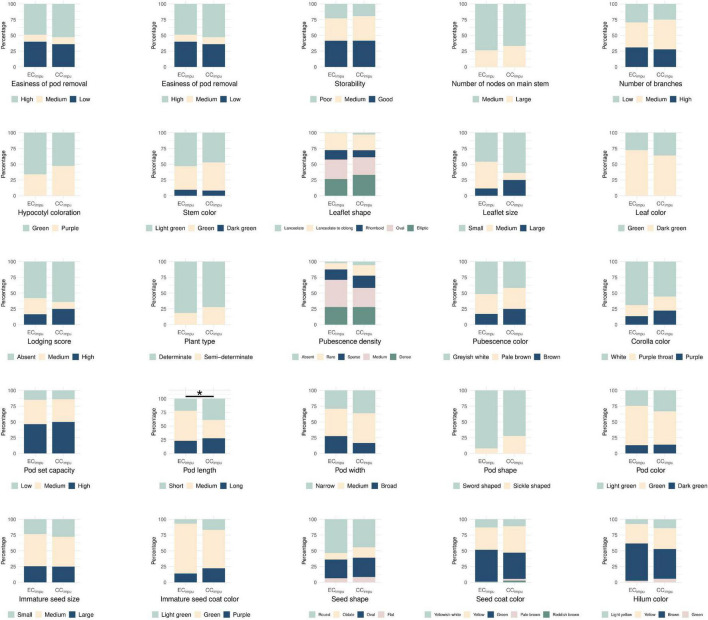
Stacked bar chart of qualitative traits in the entire collection (observed plus imputed values, EC_*impu*_) and the core collection (CC_*impu*_). Different colored bar represents specific class of phenotype. Asterisk represents significant difference between the CC_*impu*_ and the EC_*impu*_ by Chi-squared test. *p-value <0.05.

We performed difference tests, diversity comparison, cluster distribution, and assessment evaluation between the CC_*impu*_ and the EC_*impu*_ to address the representativeness of the CC_*impu*_. First of all, we compared the CC_*impu*_ to the EC_*impu*_, and found that 12 (57.14%) phenotypes retained the variability and 9 (42.86%) phenotypes had a slight loss in dispersion (Figure 4). Second, we found three imputed quantitative traits (seed thickness, from sowing to flowering, and 100 immature seed weight) and one imputed qualitative trait (pod length) showing weakly significant differences (*p*-values ranged between 0.02 and 0.04) (Figures 4, 5 and Supplementary Tables 6, 7). The results indicated a slight mean difference (8.7%) between the two collections (acceptable threshold is <20%), indicating consistent patterns of central tendency and dispersion for all phenotypes between the CC_*impu*_ and the EC_*impu*_. No significant difference was found between the CC_*raw*_ and the EC_*raw*_ (Supplementary Tables 8, 9).

**FIGURE 6:**
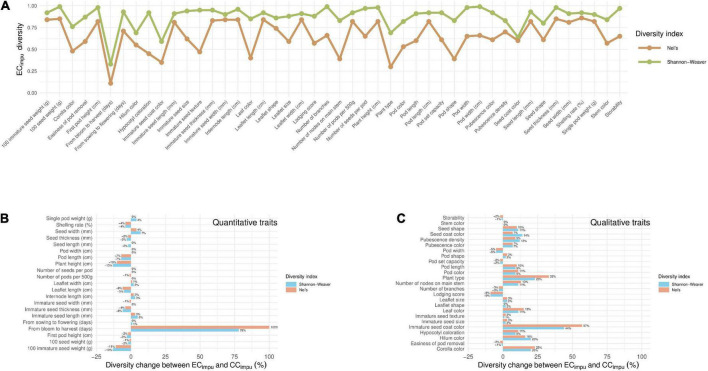
Diversity comparisons between the entire collection (observed plus imputed values, EC_*impu*_) and the core collection (CC_*impu*_) in Taiwanese vegetable soybean germplasms. **(A)** Diversity index of each phenotypic trait in the EC_*impu*_. The brown and green lines are defined as the Nei’s and Shannon–Weaver diversity indexes, respectively. **(B)** Diversity change between the EC_*impu*_ and the CC_*impu*_ in quantitative traits. **(C)** Diversity change between the EC_*impu*_ and the CC_*impu*_ in qualitative traits. The blue bar (Shannon–Weaver diversity index) and the red bar (Nei’s diversity index) mean diversity is retained (≥0%) or lost (<0%) of the CC_*impu*_ compared to the EC_*impu*_.

**FIGURE 7:**
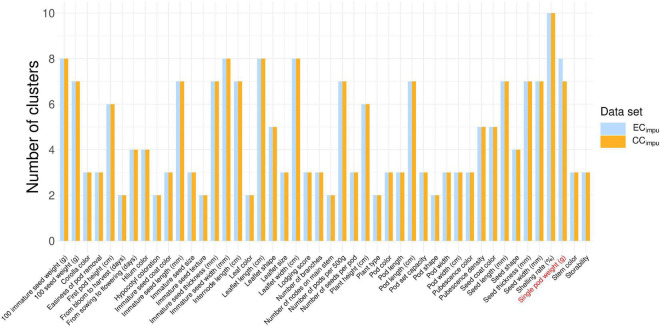
Distribution of clustering of the entire collection (observed plus imputed values, EC_*impu*_) and the core collection (CC_*impu*_). The blue and orange bars represent the number of clusters in the EC_*impu*_ and CC_*impu*_, respectively. Clustering analysis for quantitative traits was conducted using the weighted *k*-means clustering algorithm. Only the “single pod weight” trait has less number of clusters in the CC_*impu*_, representing almost full coverage from the EC_*impu*_.

The phenotypic diversity for each of traits was then compared between the EC_*impu*_ and the CC_*impu*_ (Figure 6 and Supplementary Table 10). We applied an unsupervised learning strategy through the weighted *k*-means algorithm to search for optimal clusters for each of the quantitative traits in the EC_*impu*_ (Figure 7). The optimal number of clusters is characterized by high diversity richness and evenness (Shannon–Weaver diversity index >90%, Nei’s diversity index >80%) and a large proportion of variance explained (>75%). For quantitative traits (Figure 6B), the Shannon–Weaver diversity index of the EC_*impu*_ and the CC_*impu*_ ranged from 0.33 to 0.99 and 0.58 to 0.99, with an equal average of 0.91, respectively. The Nei’s diversity index of the EC_*impu*_ and the CC_*impu*_ ranged from 0.11 to 0.86 and 0.24 to 0.85, with an overall average of 0.77 and 0.76, respectively. These suggested that more than half (or 42.85%) of the quantitative phenotypes retained (or slight lost from 1 to 13%) diversity richness and evenness. The diversity differences between the EC_*impu*_ and the CC_*impu*_ were almost equivalent. For qualitative traits (Figures 6C, 7), both the CC_*impu*_ and the EC_*impu*_ had an equal number of clusters in all traits, demonstrating 100% coverage. The Shannon–Weaver diversity index of the EC_*impu*_ and the CC_*impu*_ ranged from 0.59 to 0.99 and 0.73 to 1.00, with an overall average of 0.85 and 0.9, respectively. The Nei’s diversity index of the EC_*impu*_ and the CC_*impu*_ ranged from 0.3 to 0.74 and 0.4 to 0.76, with an overall average of 0.55 and 0.58, respectively. These suggested that more than three quarters (or 24%) of the quantitative phenotypes retained (or slightly loss from 1 to 9%) diversity richness and evenness. On the whole, diversity richness and evenness were retained and preserved well in the CC_*impu*_, indicating the our selected CC_*impu*_ is representative of diversity from the EC_*impu*_. Similar diversity preservation or loss was observed between the EC_*raw*_ and the CC_*raw*_ (please refer to Supplementary Table 11). Again, our results support the applicability of the MI-based method in exploring population structure and constructing a CC.

The CC_*impu*_ selected by PowerCore provided a perfect coverage (100% in qualitative traits and 99.4% in quantitative traits) for the entire collection (Table 3), suggesting that important phenotypic features and variability were preserved in the CC_*impu*_. Critically, the performance of small MD% (4.51%) and VD% (42.41%), and large CR% (98.10%) and VR% (138.76%) indices for the CC_*impu*_ reflected their effectiveness and good representation in capturing varying ranges of phenotypic variability in the entire collection. The assessment of the CC_*raw*_ in four indices (MD% = 4.14%, VD% = 40.65%, CR% = 96.81%, and VR% = 135.1%) was equivalent to that of the CC_*impu*_. Again, MI-based imputed phenotypes can establish a representative core set of Taiwanese vegetable soybeans.

**TABLE 3:** Evaluation of the core collection compared to the entire collection in Taiwanese vegetable soybean germplasms.

The property of the core collection	*N* (%)	MD%*^a^*	VD%*^a^*	CR%*^a^*	VR%*^a^*	Coverage*^b^*	
						Quantitative traits	Qualitative traits
CC_*impu*_	36 (18.0%)	4.51	42.41	98.10	138.76	99.40	100
CC_*raw*_	43 (21.5%)	4.14	40.65	96.81	135.10	NA	100

CC*_raw_* and CC*_impu_*, core collections selected with PowerCore using observed and complete (observed plus imputed values) phenotypes, respectively; N, number of phenotypes; (%), percentage of the CC that accounted for the EC; MD%, mean difference percentage; VD%, variance difference percentage; CR%, coincidence rate; VR%, variable rate; NA, not available.

^a^The evaluation indices of MD%, VD%, CR%, and VR% were calculated based on all 25 quantitative traits.

^b^Coverage was computed based on all 46 mixed-type phenotypic traits.

^c^The CC is considered to be the representative of the EC only when (1) MD% is no more than 20%, (2) CR% is greater than 80%, and (3) coverage is close to 100.

**FIGURE 8:**
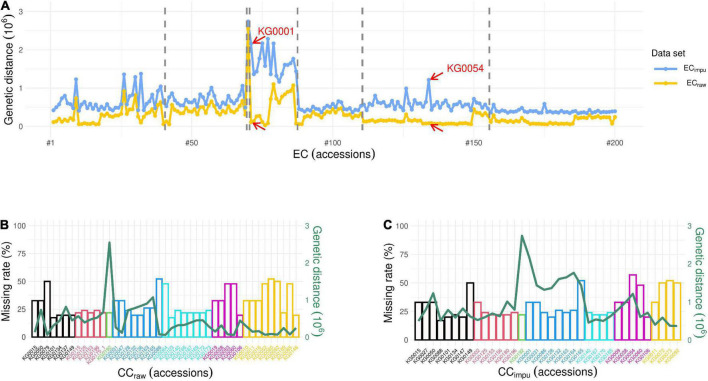
Impact of missing rate on genetic distance. **(A)** Distribution of genetic distance in seven different clusters for 200 accessions in the entire collection. The blue and yellow lines represent the genetic distance calculated using modified Roger’s distance based on complete (i.e., observed plus imputed values) phenotypes (EC_*impu*_) and observed phenotypes (EC_*raw*_), respectively. The dash line separates the distribution of genetic distance into different clusters. The red arrow pointing to accessions (KG0001 and KG0054) means they have larger difference in genetic distance between the EC_*impu*_ and the EC_*raw*_, for example. **(B)** Distribution of clustering, phenotype missing rate, and genetic distance for 43 accessions in the core collection using observed phenotypes (i.e., CC_*raw*_). **(C)** Distribution of clustering, phenotype missing rate, and genetic distance for 36 accessions in the core collection using complete phenotypes (i.e., CC_*impu*_). The *x*-axis (bottom) represents the number of clusters and cluster distribution. The *y*-axis (left) represents the missing rate (%). The secondary *y*-axis (right) represents the genetic distance. The length and marginal color of the bar represent the missing rate of each core accession and distribution of clustering, respectively. The green line represents the distribution of genetic distance of the core accessions.

To investigate the impact of missing phenotypes on the establishment of the CC, we first set four different thresholds of missing rate (0, <30, <65, and ≤100%) to evaluate the selected accessions of the CC. Table 4 demonstrates the number of phenotypic traits used to construct the CC, the overall phenotype missing rate in the EC, and the number of selected accessions in the CC under a given threshold of the missing rate. In general, CC size should be in control of about 10% of the EC, as Brown (1989b) recommended. The higher the threshold of the missing rate in phenotypic traits, the more phenotypic traits were used in the EC resulting in more numbers of core collection accessions in both the CC_*raw*_ and the CC_*impu*_. We observed the fact that the more phenotypes in the EC, the more complicated the relatedness kinship and population structure among germplasm accessions; thus, more accessions were selected to be representative of the EC. As we can see, the number of accessions in the CC (both the CC_*raw*_ and the CC_*impu*_) was increased with higher (i.e., loose) threshold of the missing rate in phenotypes. Most interestingly, the number of core accessions in the CC_*impu*_ was smaller than that in the CC_*raw*_ under all the thresholds. Second, we examined how missing rate affects the selection of the CC. Figure 8 reveals the impact of phenotype missing rate on calculating the genetic distance for the core accessions in the CC. The *x*-axis (the bottom) represents the number of clusters and cluster distribution. The *y*-axis (left) represents the missing rate (%). The secondary *y*-axis (the right) represents the genetic distance. The length and marginal color of the bar represents the missing rate of each core accession and the distribution of clustering, respectively. The red line is defined as the genetic distance calculated using modified Roger’s distance. It is clear to see that the genetic distances of the CC_*raw*_ (Figure 8B) are smaller than those of the CC_*impu*_ (Figure 8C). The underestimated genetic distances (or similarity) contributed to unpaired phenotypes because of missingness. As we can see, some accessions with a small or equivalent genetic distance may be selected by chance to be members of the CC; particularly, this situation obviously occurred in the CC_*raw*_. In addition, missingness can lead to biased estimates; under such conditions, the higher the missing rate, the smaller the genetic distance (i.e., more similar) (Figure 8B). Fortunately, this issue does not present in the CC_*impu*_ (Figure 8C). As shown in Figure 8A, there are two accessions (KG0001 and KG0054) with high genetic distance based on complete phenotypes (EC_*impu*_), while low genetic distance is revealed based on observed phenotypes (EC_*raw*_). Both of them were only included in the CC_*impu*_ (excluded in the CC_*raw*_), suggesting that they have some unique characteristic covered by incomplete data. To sum up, the MI-based core collection can capture accessions with valuable characteristics and retained variability from the EC.

**TABLE 4:** Impact of phenotype missing rate on the establishment of the core collection.

	Threshold of phenotype missing rate			
	0%	<30%	<65%	≤100%
Number of phenotypic traits in the entire collection (EC)	6	29	38	46
The overall phenotype missing rate in the EC	0%	12%	22%	31%
Number of accessions in the CC_*raw*_*^a^*(%)	14 (7.0%)	30 (15.0%)	32 (16.0%)	43 (21.5%)
Number of accessions in theCC_*impu*_*^a^*(%)	14 (7.0%)	28 (14.0%)	30 (15.0%)	36 (18.0%)

CC*_raw_*, the core collection established using observed phenotypes; CC*_impu_*, the core collection established using complete (observed plus imputed values) phenotypes; (%), percentage of the CC that accounted for the EC.

^a^CC*_raw_* and CC*_impu_* were established using PowerCore to analyze the observed (EC*_raw_*) and complete (EC*_impu_*) phenotypes, respectively.

## Discussion

Our vegetable soybean (edamame) germplasm collection contains 213 accessions and 47 phenotypic traits (morphology, growth, phenology, and production), which preserved the richest resources of diverse accessions and phenotypic diversities worldwide. Edamame is a type of specialty soybean and is harvested as immature beans and eaten as a snack or a vegetable. Unlike grain soybean, edamame is characterized by several features including large seed size, high isoflavone content, cold tolerance, higher moisture content, stay-green pods, and sweet and delicate flavor. Edamame is primarily grown during the autumn and spring seasons in Taiwan’s Kao-Ping and Yun-Chia-Nan areas.

Soybeans (including edamame) have been cultivated in Taiwan for several decades. Before the 1970s, the edamame market in Taiwan was dominated by fresh shelled beans. During 1969–1970, several varieties introduced from Japan were especially chosen for planting, and of which two specific varieties were processed into frozen edamame for export. After that, fresh frozen edamame has become an essentially popular snack in Taiwan’s market. With the improvement in freezing equipment, processing chain, technology development, and safety management system, the time to harvest edamame has been shortened. Improved edamame varieties produce better freshness and improved quality taste. To date, Taiwan’s edamame with multiple unique commercial varieties has been successfully sold in international markets around the world. Therefore, edamame is known as “Taiwan’s green gold.”

Over recent decades, the awakening of dietary and healthy eating habits has promoted the consumption and development of edamame in the United States. From 2000 to 2008, there was a 300% increase in consumption of edamame in the United States (Sams et al., 2012). With the current trend, the demand for edamame will continue to increase (Zhang et al., 2017). However, most of the edamame consumed was imported from China. The development and improvement of edamame in the United States are relatively late because of some limitations including poor phenotypic and genetic resources, poor seed germination, poor seedling emergence and establishment, susceptibility to seed diseases, and others (Jiang G. L. et al., 2018). The situations mentioned above brought more attention to the potential of vegetable soybean germplasms.

In our vegetable soybean germplasm accessions, a scenario of multiple correlated phenotypes (Figure 1 and Supplementary Table 1) and missing phenotypes (Supplementary Tables 2, 3) was observed. This central issue is typically seen in related samples and can lead to many statistical problems (Dahl et al., 2016). For instance, many methods such as principle component analysis and clustering analysis were developed mainly for complete (without missing values) multiple phenotypes. Missing phenotypes in germplasms often reduce sample sizes (i.e., number of accessions) and result in significant loss of power and misunderstanding of the genetic architecture of complex multiple correlated phenotypes. In particular, substantial missing phenotypes across accessions may produce no samples with completely observed phenotypes. This scenario also occurs in our vegetable soybean germplasm because of cultivation problems, negligent investigations, and environmental conditions. Although missing phenotypes are pervasive in germplasm accessions and large accessions are often difficult to collect, little is known about the detrimental impact of missingness on the power to explore the whole map of population structure, kinship relatedness, and genetic diversity of germplasms.

Missing data may increase uncertainty in vegetable soybean germplasms and cause inaccurate analysis results. Especially, the CC established by multiple correlated phenotypes really relies on data completeness. In this study, we applied a model-based imputation algorithm, multiple phenotype imputation, through Bayesian linear regression computationally to impute missing phenotypes. The uncertainty arising from the imputation procedure was considered to be minimized (Lee and Simpson, 2014). Through the multiple phenotype imputation process, missing phenotypes were imputed by repeatedly sampling from a fitted imputation model (the first stage) and then by averaging the estimates derived from each individual of complete datasets (the second stage). Hence, the multiple phenotype imputation method is able to account for the uncertainty within and across the complete (i.e., observed plus imputed phenotypes) datasets due to the missingness (Lee and Simpson, 2014). We noticed that a slight change in correlation structure (i.e., marginally decreased correlations) was present in our imputed phenotypes, which is typically seen in imputed datasets (Taylor et al., 2017). Although none of the imputation methods can perfectly preserve the original correlation structure of phenotypes, the MI method is beneficial in terms of bias and uncertainty (i.e., reduction in errors) and outperforms other methods such as average-based imputation and single imputation (Taylor et al., 2017; Madley-Dowd et al., 2019). Most importantly, the multiple phenotype imputation method provides less errors and gains in accuracy. In particular, the MI method works best when the data missing rate is high or the sample size is medium ranging between 50 and 1,000 regardless of missing rate, compared to other missing data imputation methods (Cheema, 2014). Therefore, the MI method can serve as the most efficient and robust method for handling missing phenotypes.

The degree of potential biases caused by missingness really depends on mechanisms underlying missing data and approaches to deal with missing data (Jakobsen et al., 2017). The MI method can be applied to many kinds of data, including phenotypes and genotypes (Soley-Bori, 2013). Several success examples in plant and human studies, for instance Plant-Impute DB (Gao et al., 2021), imputed low-density marker chip data in plant breeding (Niehoff et al., 2022) and GWAS genotypes in rice (Wang et al., 2018). We noticed that the MI method outperforms the single imputation methods (e.g. average-based approaches). The former has unbiased and accurate estimates, and works computationally efficient. In particular, the MI method using the Bayesian model performed better with slightly higher accuracy than that using the non-Bayesian model (Tian et al., 2015). Efficient utilization of germplasm resources is really a challenging task for plant-breeding. A precise and accurate CC can help breeders and scientists in reducing breeding program workload (van Hintum et al., 2000).

The chance of at least one observation being missed increases exponentially as the number of phenotypes increases. This situation also occurred in our study. We observed a dramatic increase in overall missing rate, from 12 to 22% and then to 31%, for all the traits in the corresponding EC as phenotypic traits increased from 29 to 38% and then to 46%, respectively (Table 4). High overall missing rate can slightly affect the results of difference tests between the EC_*raw*_ and the EC_*impu*_ (Supplementary Tables 4, 5). In this study, we found four (8%) phenotypic traits that reached a significant difference because of high missing rate (more than 50.5%) and/or only two possible classes in a trait, which is acceptable and negligible. This is often observed in complex correlated phenotypes in related samples (Dahl et al., 2016).

Core collections (CCs) have a small size to promote breeder screening and improve cultivars (Frankel, 1984; Brown, 1989b). In general, a CC should have 10% of the entire collection (EC) size and represent 70% of the genetic diversity at least of the EC (Brown, 1989a). During the process, we used the PowerCore v1.0 software to construct the CC_*impu*_ and CC_*raw*_ from the EC_*impu*_ and EC_*raw*_, respectively. Both the CC_*impu*_ and the CC_*raw*_ exhibited a small mean difference percentage (both 4.51 and 4.14% were less than the significance critical value of 20%) and a variance difference percentage (42.41 and 40.65%). However, high coincidence rate (both 98.1 and 96.81% were higher than the critical value of 80%) and variable rate (138.76 and 135.10%) were also noted, indicating a wide range of diversity in phenotypic variability in the CC compared to the EC (Table 3). Both the CC_*impu*_ and the CC_*raw*_ showed a perfect coverage of 100% on the qualitative traits, suggesting they contain all types of qualitative traits. We found a nearly perfect coverage (99.40%) on the quantitative traits in the CC_*impu*_. However, PowerCore did not provide coverage on quantitative traits in the CC_*raw*_ because of missingness in the phenotypes.

Missingness can seriously affect the selection of core accessions. In addition, CC selection may tend to choose accessions with low missing rate. For instance, there are 18 accessions with low missing rate (<25%) selected in the CC_*raw*_ compared to the 23 accessions selected in the CC_*impu*_ (Figures 8B,C). The CC_*raw*_ have nine accessions (21%) in the seventh cluster, while the CC_*impu*_ have only four accessions (11%) in the seventh cluster but have more accessions (22%) in the fourth cluster. In contrast to the accessions in the seventh cluster, the accessions in the fourth cluster have an obvious difference genetic distance between EC_*raw*_ and EC_*impu*_, suggesting that germplasms have some unknown phenotypes and are unable to reflect the actual morphological variation of populations. Accurate methods for imputing missing data may also be helpful in capturing underlying patterns of real variation (Stephens and Scheet, 2005). Furthermore, the overall phenotypic diversity in the CC_*impu*_ was equal to that in the CC_*raw*_ (Table 1). This suggests that the MI-based method can be an efficiently reliable way to boost power and preserve higher diversity in less core collection accessions.

From diversity comparisons between the core collection and the complete entire collection (Figure 6 and Supplementary Table 10), 28 (61%) traits retained phenotypic diversity, and 16 (35%) had up to 9% diversity loss. Only two traits (plant height and 100 immature seed weight) lost diversity by more than 10% (up to 13%) in both diversity indices, suggesting that the CC_*impu*_ retained the high diversity and evenness of the EC_*impu*_. A Venn diagram and five indices (MD%, VD%, CR%, VR%, and coverage) of the CC_*impu*_ and CC_*raw*_ are given in Supplementary Figure 1 and Supplementary Table 12. Using complete phenotypes demonstrated better properties of five indices (i.e., good representation of genetic diversity) of the EC in the intersection (21 accessions) of the CC_*raw*_ and the CC_*impu*_ compared to the use of observed phenotypes. Nevertheless, the CR% in both the difference of the sets CC_*raw*_ and CC_*impu*_ (denoted with CC_*raw*_ and CC_*impu*_) and the difference between the sets CC_*impu*_ and CC_*raw*_ (denoted with CC_*impu*_ and CC_*raw*_) was less than the threshold of 80%. Compared to the results of the CC_*impu*_ and CC_*raw*_ (Table 3), it is worth noting that the 21 core accessions are prioritized to be used for breeding programs.

Kao et al. (2021) proposed a modified Roger’s distance algorithm to construct a CC based on 29 phenotypic traits of Taiwanese vegetable soybean germplasms. We found that sixteen accessions overlapped between the CC_*impu*_ and our previous results (please refer to Table 2). Among the sixteen accessions, KG0132 and KG0101 have the longest immature seed length and thickest immature seeds, respectively. Besides, both have a “large” characteristic in terms of immature seed size, suggesting that the CC retained valuable edamame traits of the immature seeds. The variety KG0031 recorded only 17.3 cm mean plant height and 4.3 cm mean first pod height; both values are smaller than the overall average minus the standard deviation of all accessions of the traits. However, low plant height and first pod height restricted its production and it was not suitable for mechanical harvesting (Zdravković et al., 2005; Jiang H. et al., 2018). However, KG0031 had a purple corolla color, which means it may have more flavonoids (such as dihydroflavonols) (Iwashina et al., 2007; Takahashi et al., 2017). Overall, many accessions of the CC_*impu*_ are in accordance with findings reported by Kao et al. (2021), indicating two algorithms (advanced M strategy on PowerCore and modified Roger’s distance) are useful to construct the CC with maximal representative and high diversity from the EC.

Among the CC_*impu*_ (Table 2), Kou et al. (2000) revealed that KG0180 (Yukinoshita-28) has the highest friability and the lowest adhesion in all 30 varieties concerned by conducting a texture profile analysis. Another extremely early maturity accession called KG0073 (Gokuwase Hayabusa) has high phenolic compounds, which represent the rich contents of alcohol- and water-soluble antioxidants and sulfur-containing amino acids (Kaizuma et al., 1974; Shafigullin et al., 2020). By comparing our results to those of previous studies, some of our CC_*impu*_ have elite characteristics that are also useful for breeding programmers and researchers.

**FIGURE 9:**
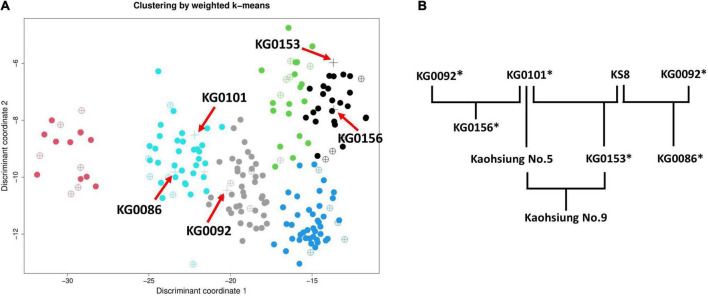
Cluster analysis and tree diagram of five crucial vegetable soybean accessions in Taiwan breeding programs. **(A)** For simplicity, weighted *k*-means clustering was performed on 199 accessions (except for the outlier “Mikowashima”) using 46 complete phenotypic traits. The red arrow pointing to the plus (+) symbol represents the five accessions (KG0086, KG0092, KG0101, KG0153, and KG0156). **(B)** History of development processes of vegetable soybean varieties in Taiwan. Accessions with the asterisk (*) symbol were incorporated in the CC_*impu*_.

In the early 1980s, crop research institutes in Taiwan got on with the improvement work for vegetable soybean (please refer to Figure 9B). In order to meet the needs of domestic and export demands, a high-yielding variety, “KG0156,” derived from a cross between KG0092 (from South Korea, used as the female parent) and KG0101 (from Japan, used as the male parent) was selected and developed in the AVRDC in Taiwan (Shanmugasundaram et al., 1991). In 1991, two new varieties, KG0153 (KG0101 × KS8) and KG0086 (KG0092 × KS8), were released by KDARES to farmers (Cheng, 1993; Cheng and Chen, 1993) and became popular varieties for exporting at that time. To this day, Kaohsiung No. 9, one of the most important vegetable soybean cultivars in Taiwan, is being selected from a cross between Kaohsiung No. 5 (derived from the pure line population of KG0101) and KG0153 (Chen and Cheng, 1996; Chou, 2008). Compared to their parental line Kaohsiung No. 5, Kaohsiung No. 9 displayed better performances including higher yield, suitability for mechanical harvesting, larger immature seeds, and higher isoflavone content (total isoflavones: 2,131 μg/g). Nowadays, Kaohsiung No. 9 has become the dominant variety for export of frozen edamame.

As shown in Figure 9A, the five crucial accessions in Taiwanese breeding programs are dispersed in three clusters. The clusters of all improved varieties (KG0086, KG0153, and KG0156) differ from their known parents (KG0092 and KG0101). Nevertheless, KG0153 and KG0156 were classified into the same cluster, and they share the same parent, “KG0101.” In terms of phenotypic traits, seed length, seed width, seed thickness, 100 seed weight, and 100 immature seed weight were shown to be highly related to “high yield” in vegetable soybean (Panthee et al., 2005; Sun et al., 2012; Hu et al., 2013; Yang et al., 2013). Except for KG0086, the other accessions (KG0092, KG0101, KG0153, and KG0156) have seed length of 9.3-11 mm, seed width of 8.7–9.7 mm, seed thickness of 6.8–7.6 mm, and 100 seed weight of 42.7–46 g. As for 100 immature seed weight, KG0086 is the best with up to 83 g. The findings confirmed that Taiwanese breeders had selected suitable germplasms to meet the breeding objective.

In conclusion, the selected core accessions in the CC_*impu*_ involving the parents of Taiwanese commercial varieties (please refer to Figure 9 and Table 2) were a significant contribution to developing commercial edamame varieties in Taiwan. Furthermore, the history of the breeding program of vegetable soybean in Taiwan provides evidence to prove that our CC_*impu*_ is helpful for breeders to screen distinguished breeding materials. Besides, accessions with desirable traits in the CC_*impu*_ identified in previous studies may also be considered promising materials for future crop improvement programs.

## Conclusion

Taiwan preserves considerable vegetable soybean germplasm accessions, and is a forerunner in the field of edamame breeding and improvement. We conducted multiple phenotype imputation to demonstrate the effectiveness and reliability of the imputed phenotypic data in exploring genetic diversity and constructing the CC. Our results showed that missingness can bias genetic distance and diversity calculation, which results in non-precise selection of the CC. In addition, the size of the CC_*raw*_ (building the CC without MI approach) is larger than that of the CC_*impu*_ (building the CC with MI approach), suggesting that workloads in a breeding program would be heavier. The CC_*impu*_ showed small mean difference and variance difference and high coincidence rate and coverage, suggesting well representativeness of the whole germplasms. Besides, some unique characteristics in our CC_*impu*_ may contribute to the development of new commercial varieties. Facing with challenges of missing phenotypes, the MI-based imputed phenotypes could be a solution to select core accessions from the entire collection efficiently.

## Data availability statement

The original contributions presented in this study are included in the article/Supplementary material, further inquiries can be directed to the corresponding author.

## Author contributions

C-FK: study conception and design. Y-HH, C-FK, and C-AW: acquisition and analysis of data. C-FK, Y-HH, C-AW, SC, P-CL, and P-YJ: interpretation of data. Y-HH, C-FK, H-MK, L-YC, C-AW, and S-SH: drafting of manuscript. C-FK, Y-HH, and H-MK: revision of the manuscript. All authors read and approved the final version of the manuscript.
