# Supplementary material for: A multiple phenotype imputation method for genetic diversity and core collection in Taiwanese vegetable soybean
Source: Front Plant Sci. 2022 Sep 2;13:948349. doi: 10.3389/fpls.2022.948349 (PMC9480828; doi:10.3389/fpls.2022.948349)

**Supplementary Table 1.** Correlation coefficient among 46 traits for observed phenotypes and complete (observed plus imputed values) phenotypes.

|  | Correlation coefficient | | |  | Correlation test | |
| --- | --- | --- | --- | --- | --- | --- |
|  | Coefficient range | Correlation strength | Pairs of phenotype |  | Result | Pairs of phenotype |
| **EC_raw_** |  |  |  |  |  |  |
|  | -0.7 to -1.0 | Very strong | 1 |  | Significant correlation | 327 |
|  | -0.5 to -0.7 | Strong | 1 |  | Non-significant correlation | 524 |
|  | -0.3 to -0.5 | Moderate | 51 |  | Not available | 111 |
|  | 0 to -0.3 | Weak | 341 |  |  |  |
|  | 0 | None | 0 |  |  |  |
|  | 0 to 0.3 | Weak | 382 |  |  |  |
|  | 0.3 to 0.5 | Moderate | 77 |  |  |  |
|  | 0.5 to 0.7 | Strong | 16 |  |  |  |
|  | 0.7 to 1.0 | Very strong | 10 |  |  |  |
|  | Not available |  | 111 |  |  |  |
| **EC_impu_** |  |  |  |  |  |  |
|  | -0.7 to -1.0 | Very strong | 0 |  | Significant correlation | 426 |
|  | -0.5 to -0.7 | Strong | 1 |  | Non-significant correlation | 564 |
|  | -0.3 to -0.5 | Moderate | 30 |  | Not available | 0 |
|  | 0 to -0.3 | Weak | 436 |  |  |  |
|  | 0 | None | 0 |  |  |  |
|  | 0 to 0.3 | Weak | 464 |  |  |  |
|  | 0.3 to 0.5 | Moderate | 45 |  |  |  |
|  | 0.5 to 0.7 | Strong | 9 |  |  |  |
|  | 0.7 to 1.0 | Very strong | 5 |  |  |  |
|  | Not available |  | 0 |  |  |  |
| Abbreviation: EC_raw_, observed entire collection; EC_impu_, complete (observed plus imputed values) entire collection. | | | | | | |

**Supplementary Table 2.** Summary information of category and missing rate in all 21 quantitative traits.

| **Quantitative traits** | | | |
| --- | --- | --- | --- |
| **Category** | **Phenotypic traits** | **N** | **Missing rate (%)^a^** |
| Morphology |  |  |  |
|  | Seed length (mm) | 200 |  |
|  | Seed width (mm) | 200 |  |
|  | Seed thickness (mm) | 200 |  |
|  | Leaflet length (cm) | 200 |  |
|  | Leaflet width (cm) | 200 |  |
|  | Pod length (cm) | 149 | 25.5 |
|  | Pod width (cm) | 149 | 25.5 |
|  | Single pod weight (g) | 99 | 50.5 |
|  | Number of pods per 500g | 99 | 50.5 |
|  | Number of seeds per pod | 50 | 75.0 |
|  | Shelling rate (%) | 150 | 25.0 |
|  | Immature seed length (mm) | 149 | 25.5 |
|  | Immature seed width (mm) | 150 | 25.0 |
|  | Immature seed thickness (mm) | 150 | 25.0 |
| Growth |  |  |  |
|  | Internode length (cm) | 50 | 75.0 |
|  | Plant height (cm) | 198 | 1.0 |
|  | First pod height (cm) | 150 | 25.0 |
| Phenology |  |  |  |
|  | From sowing to flowering (days) | 49 | 75.5 |
|  | From bloom to harvest (days) | 43 | 78.5 |
| Production |  |  |  |
|  | 100 seed weight (g) | 200 |  |
|  | 100 immature seed weight (g) | 149 | 25.5 |
| Abbreviation: N, number of accessions.  ^a^Missing rates were calculated based on 200 accessions. | | | |

**Supplementary Table 3.** Summary information of category and missing rate in all 25 qualitative traits.

| **Qualitative traits** | | | |
| --- | --- | --- | --- |
| **Category** | **Phenotypic traits** | **N** | **Missing rate (%)^a^** |
| Morphology |  |  |  |
|  | Seed shape | 199 | 0.5 |
|  | Seed color | 189 | 5.5 |
|  | Hilum color | 177 | 11.5 |
|  | Hypocotyl coloration | 199 | 0.5 |
|  | Number of nodes on main stem | 99 | 50.5 |
|  | Stem color | 182 | 9.0 |
|  | Number of branches | 199 | 0.5 |
|  | Leaflet size | 150 | 25.0 |
|  | Leaflet shape | 147 | 26.5 |
|  | Leaf color | 150 | 25.0 |
|  | Pubescence density | 199 | 0.5 |
|  | Pubescence color | 197 | 1.5 |
|  | Corolla color | 199 | 0.5 |
|  | Pod set capacity | 149 | 25.5 |
|  | Pod length | 95 | 52.5 |
|  | Pod width | 98 | 51.0 |
|  | Pod shape | 99 | 50.5 |
|  | Pod color | 48 | 76.0 |
|  | Immature seed size | 99 | 50.5 |
|  | Immature seed coat color | 99 | 50.5 |
|  | Immature seed texture | 50 | 75.0 |
|  | Easiness of pod removal | 50 | 75.0 |
|  | Storability | 50 | 75.0 |
| Growth |  |  |  |
|  | Lodging score | 71 | 64.5 |
|  | Plant type | 149 | 25.5 |
| Abbreviation: N, number of accessions.  ^a^Missing rates were calculated based on 200 accessions. | | | |

**Supplementary Table 4.** Difference tests of quantitative traits between observed phenotypes and complete (observed plus imputed values) phenotypes.

| **Phenotypic trait** | **EC_raw_** | |  | **EC_impu_**^a^ | |  | | **Difference test (*p*-value)^b^** |
| --- | --- | --- | --- | --- | --- | --- | --- | --- |
|  | **N** | **Mean ± s.d.** |  | **N** | **Mean ± s.d.** | |  |  |
| Seed length (mm) | 200 | 8.96 ± 0.78 |  | 200 | 8.96 ± 0.78 | |  | 1.00 |
| Seed width (mm) | 200 | 8.28 ± 0.59 |  | 200 | 8.28 ± 0.59 | |  | 1.00 |
| Seed thickness (mm) | 200 | 7.10 ± 0.67 |  | 200 | 7.10 ± 0.67 | |  | 1.00 |
| 100 seed weight (g) | 200 | 33.41 ± 7.51 |  | 200 | 33.41 ± 7.51 | |  | 1.00 |
| Internode length (cm) | 50 | 3.79 ± 0.99 |  | 200 | 3.59 ± 0.82 | |  | 0.14 |
| Plant height (cm) | 198 | 37.09 ± 10.71 |  | 200 | 36.97 ± 10.74 | |  | 0.90 |
| Leaflet length (cm) | 200 | 10.60 ± 7.61 |  | 200 | 10.60 ± 7.61 | |  | 1.00 |
| Leaflet width (cm) | 200 | 7.22 ± 1.18 |  | 200 | 7.22 ± 1.18 | |  | 1.00 |
| From sowing to flowering (days) | 49 | 27.45 ± 1.77 |  | 200 | 27.31 ± 1.60 | |  | 0.58 |
| From bloom to harvest (days) | 43 | 66.37 ± 1.73 |  | 200 | 66.52 ± 1.29 | |  | **0.004** |
| Pod length (cm) | 149 | 4.59 ± 0.53 |  | 200 | 4.61 ± 0.50 | |  | 0.64 |
| Pod width (cm) | 149 | 1.22 ± 0.28 |  | 200 | 1.22 ± 0.24 | |  | 0.85 |
| Single pod weight (g) | 99 | 2.10 ± 0.49 |  | 200 | 2.14 ± 0.44 | |  | 0.44 |
| Number of pods per 500g | 99 | 187.83 ± 33.51 |  | 200 | 186.82 ± 33.24 | |  | 0.81 |
| First pod height (cm) | 150 | 11.85 ± 4.41 |  | 200 | 11.53 ± 4.30 | |  | 0.49 |
| Number of seeds per pod | 50 | 2.70 ± 0.86 |  | 200 | 2.95 ± 0.88 | |  | 0.08 |
| Shelling rate (%) | 150 | 56.79 ± 6.97 |  | 200 | 57.16 ± 7.56 | |  | 0.65 |
| Immature seed length (mm) | 149 | 15.49 ± 1.63 |  | 200 | 15.54 ± 1.55 | |  | 0.78 |
| Immature seed width (mm) | 150 | 11.04 ± 3.13 |  | 200 | 10.97 ± 2.81 | |  | 0.81 |
| Immature seed thickness (mm) | 150 | 8.07 ± 0.82 |  | 200 | 8.06 ± 0.83 | |  | 0.88 |
| 100 immature seed weight (g) | 149 | 69.05 ± 14.87 |  | 200 | 68.67 ± 14.62 | |  | 0.81 |
| Abbreviation: EC_raw_, observed entire collection; EC_impu_, complete (observed plus imputed values) entire collection; N, number of germplasms; s.d., standard deviation.  ^a^Multiple phenotypes imputation was used to estimate missing phenotypes. ^b^Student *t*-test was conducted to test difference among EC_raw_ and EC_impu_ (the *p*-value smaller than 0.05 is highlighted in bold). | | | | | | | | |

**Supplementary Table 5.** Difference tests of qualitative traits between observed phenotypes and complete (observed plus imputed values) phenotypes.

| **Phenotypic trait** | **EC_raw_** | |  | **EC_impu_^a^** | |  | **Difference test**  **(*p*-value)^b^** |
| --- | --- | --- | --- | --- | --- | --- | --- |
|  | **N** | **(%)** |  | **N** | **(%)** |  |  |
| Seed shape |  |  |  |  |  |  | 1.00 |
| Round | 107 | (53.8) |  | 107 | (53.5) |  |  |
| Oblate | 21 | (10.5) |  | 21 | (10.5) |  |  |
| Oval | 58 | (29.2) |  | 59 | (29.5) |  |  |
| Flat | 13 | (6.5) |  | 13 | (6.5) |  |  |
| Seed coat color |  |  |  |  |  |  | 1.00 |
| Yellowish white | 26 | (13.8) |  | 26 | (13.0) |  |  |
| Yellow | 69 | (36.5) |  | 71 | (35.5) |  |  |
| Green | 92 | (48.7) |  | 101 | (50.5) |  |  |
| Pale brown | 1 | (0.5) |  | 1 | (0.5) |  |  |
| Reddish brown | 1 | (0.5) |  | 1 | (0.5) |  |  |
| Hilum color |  |  |  |  |  |  | 0.99 |
| Light yellow | 13 | (7.3) |  | 15 | (7.5) |  |  |
| Yellow | 58 | (32.8) |  | 62 | (31.0) |  |  |
| Brown | 102 | (57.6) |  | 118 | (59.0) |  |  |
| Green | 4 | (2.3) |  | 5 | (2.5) |  |  |
| Hypocotyl coloration |  |  |  |  |  |  | 1.00 |
| Green | 131 | (65.8) |  | 132 | (66.0) |  |  |
| Purple | 68 | (34.2) |  | 68 | (34.0) |  |  |
| Number of nodes on main stem |  |  |  |  |  |  | **0.0008** |
| Medium | 90 | (90.9) |  | 147 | (73.5) |  |  |
| Large | 9 | (9.1) |  | 53 | (26.5) |  |  |
| Stem color |  |  |  |  |  |  | 0.14 |
| Light green | 106 | (58.2) |  | 106 | (53.0) |  |  |
| Green | 68 | (37.4) |  | 75 | (37.5) |  |  |
| Dark green | 8 | (4.4) |  | 19 | (9.5) |  |  |
| Number of branches |  |  |  |  |  |  | 1.00 |
| Low | 58 | (29.1) |  | 59 | (29.5) |  |  |
| Medium | 79 | (39.7) |  | 79 | (39.5) |  |  |
| High | 62 | (31.2) |  | 62 | (31.0) |  |  |
| Lodging score |  |  |  |  |  |  | **<0.0001** |
| Absent | 64 | (90.1) |  | 116 | (58.0) |  |  |
| Medium | 6 | (8.5) |  | 51 | (25.5) |  |  |
| High | 1 | (1.4) |  | 33 | (16.5) |  |  |
| Leaflet size |  |  |  |  |  |  | 0.23 |
| Small | 81 | (54.0) |  | 92 | (46.0) |  |  |
| Medium | 58 | (38.7) |  | 85 | (42.5) |  |  |
| Large | 11 | (7.3) |  | 23 | (11.5) |  |  |
| Leaflet shape |  |  |  |  |  |  | 0.99 |
| Lanceolate | 1 | (0.7) |  | 1 | (0.5) |  |  |
| Lanceolate to oblong | 37 | (25.2) |  | 54 | (27.0) |  |  |
| Rhomboid | 24 | (16.3) |  | 30 | (15.0) |  |  |
| Oval | 46 | (31.3) |  | 62 | (31.0) |  |  |
| Elliptic | 39 | (26.5) |  | 53 | (26.5) |  |  |
| Leaf color |  |  |  |  |  |  | 0.6 |
| Green | 46 | (30.7) |  | 55 | (27.5) |  |  |
| Dark green | 104 | (69.3) |  | 145 | (72.5) |  |  |
| Plant type |  |  |  |  |  |  | **0.0002** |
| Determinate | 142 | (95.3) |  | 163 | (81.5) |  |  |
| Semi-determinate | 7 | (4.7) |  | 37 | (18.5) |  |  |
| Pubescence density |  |  |  |  |  |  | 1.00 |
| Absent | 4 | (2.0) |  | 5 | (2.5) |  |  |
| Rare | 20 | (10.1) |  | 20 | (10.0) |  |  |
| Sparse | 33 | (16.6) |  | 33 | (16.5) |  |  |
| Medium | 86 | (43.2) |  | 86 | (43.0) |  |  |
| Dense | 56 | (28.1) |  | 56 | (28.0) |  |  |
| Pubescence color |  |  |  |  |  |  | 0.98 |
| Greyish white | 102 | (51.8) |  | 103 | (51.5) |  |  |
| Pale brown | 63 | (32.0) |  | 63 | (31.5) |  |  |
| Brown | 32 | (16.2) |  | 34 | (17.0) |  |  |
| Corolla color |  |  |  |  |  |  | 0.99 |
| White | 138 | (69.4) |  | 138 | (69.0) |  |  |
| Purple throat | 34 | (17.0) |  | 35 | (17.5) |  |  |
| Purple | 27 | (13.6) |  | 27 | (13.5) |  |  |
| Pod set capacity |  |  |  |  |  |  | 0.13 |
| Low | 13 | (8.7) |  | 30 | (15.0) |  |  |
| Medium | 54 | (36.3) |  | 77 | (38.5) |  |  |
| High | 82 | (55.0) |  | 93 | (46.5) |  |  |
| Pod length |  |  |  |  |  |  | 0.59 |
| Short | 16 | (16.8) |  | 44 | (22.0) |  |  |
| Medium | 56 | (59.0) |  | 110 | (55.0) |  |  |
| High | 23 | (24.2) |  | 46 | (23.0) |  |  |
| Pod width |  |  |  |  |  |  | 0.56 |
| Narrow | 23 | (23.5) |  | 58 | (29.0) |  |  |
| Medium | 44 | (44.9) |  | 87 | (43.5) |  |  |
| Broad | 31 | (31.6) |  | 55 | (27.5) |  |  |
| Pod shape |  |  |  |  |  |  | **0.0004** |
| Sword shaped | 91 | (92.0) |  | 147 | (73.5) |  |  |
| Sickle shaped | 8 | (8.0) |  | 53 | (26.5) |  |  |
| Pod color |  |  |  |  |  |  | **0.0012** |
| Light green | 2 | (4.2) |  | 49 | (24.5) |  |  |
| Green | 43 | (89.6) |  | 125 | (62.5) |  |  |
| Dark green | 3 | (6.2) |  | 26 | (13.0) |  |  |
| Immature seed size |  |  |  |  |  |  | 0.84 |
| Small | 22 | (22.2) |  | 47 | (23.5) |  |  |
| Medium | 54 | (54.6) |  | 102 | (51.0) |  |  |
| Large | 23 | (23.2) |  | 51 | (25.5) |  |  |
| Immature seed coat color |  |  |  |  |  |  | 0.03 |
| Light green | 7 | (7.1) |  | 14 | (7.0) |  |  |
| Green | 88 | (88.9) |  | 158 | (79.0) |  |  |
| Purple | 4 | (4.0) |  | 28 | (14.0) |  |  |
| Immature seed texture |  |  |  |  |  |  | **<0.0001** |
| Soft | 3 | (6.0) |  | 75 | (37.5) |  |  |
| Slightly soft | 47 | (94.0) |  | 125 | (62.5) |  |  |
| Easiness of pod removal |  |  |  |  |  |  | 0.16 |
| High | 32 | (64.0) |  | 98 | (49.0) |  |  |
| Medium | 4 | (8.0) |  | 22 | (11.0) |  |  |
| Low | 14 | (28.0) |  | 80 | (40.0) |  |  |
| Storability |  |  |  |  |  |  | 0.65 |
| Poor | 9 | (18.0) |  | 46 | (23.0) |  |  |
| Medium | 17 | (34.0) |  | 71 | (35.5) |  |  |
| Good | 24 | (48.0) |  | 83 | (41.5) |  |  |
| Abbreviation: EC_raw_, observed entire collection; EC_impu_, complete (observed plus imputed values) entire collection; N, number of germplasms; (%), the percentage of each types accounted for the trait.  ^a^Multiple phenotypes imputation was used to estimate missing phenotypes. ^b^Chi-squared test was conducted to test difference among EC_raw_ and EC_impu_ (the *p*-value smaller than 0.05 is highlighted in bold). | | | | | | | |

**Supplementary Table 6.** Difference tests of quantitative traits between the core collection and the complete entire collection in Taiwanese vegetable soybean germplasms.

| **Phenotypic trait** | **Complete entire collection (EC_impu_)** | | | | | | |  | | **Core collection (CC_impu_)^a^** | | | | | | |  | **Difference test^b^** |
| --- | --- | --- | --- | --- | --- | --- | --- | --- | --- | --- | --- | --- | --- | --- | --- | --- | --- | --- |
|  | **N** | **Min** | **Max** | **Range** | **Mean** | **SD** | **CV(%)** |  | **N** | | **Min** | **Max** | **Range** | **Mean** | **SD** | **CV(%)** |  | ***p*-value** |
| Seed length (mm) | 200 | 7.1 | 11.2 | 4.1 | 9.0 | 0.8 | 8.7 |  | 36 | | 7.2 | 11.0 | 3.8 | 8.8 | 1.1 | 12.1 |  | 0.47 |
| Seed width (mm) | 200 | 6.2 | 9.7 | 3.5 | 8.3 | 0.6 | 7.2 |  | 36 | | 6.3 | 9.7 | 3.4 | 8.1 | 0.8 | 9.8 |  | 0.18 |
| Seed thickness (mm) | 200 | 5.1 | 8.8 | 3.7 | 7.1 | 0.7 | 9.4 |  | 36 | | 5.1 | 8.8 | 3.7 | 6.8 | 0.8 | 11.6 |  | **0.04** |
| 100 seed weight (g) | 200 | 4.2 | 51.2 | 47.0 | 33.4 | 7.5 | 22.5 |  | 36 | | 4.2 | 51.0 | 46.8 | 31.3 | 10.8 | 34.4 |  | 0.27 |
| Internode length (cm) | 200 | 1.7 | 7.0 | 5.3 | 3.6 | 0.8 | 22.9 |  | 36 | | 1.7 | 7.0 | 5.3 | 3.8 | 1.0 | 27.3 |  | 0.14 |
| Plant height (cm) | 200 | 17.3 | 70.7 | 53.4 | 37.0 | 10.7 | 29.1 |  | 36 | | 17.3 | 70.7 | 53.4 | 39.6 | 12.6 | 31.8 |  | 0.20 |
| Leaflet length (cm) | 200 | 7.0 | 116.0 | 109.0 | 10.6 | 7.6 | 71.8 |  | 36 | | 7.2 | 116.0 | 108.8 | 13.4 | 17.7 | 132.2 |  | 0.12 |
| Leaflet width (cm) | 200 | 1.4 | 10.4 | 9.0 | 7.2 | 1.2 | 16.4 |  | 36 | | 1.4 | 10.4 | 9.0 | 7.2 | 1.6 | 22.6 |  | 0.79 |
| From sowing to flowering (days) | 200 | 25.0 | 32.0 | 7.0 | 27.3 | 1.6 | 5.9 |  | 36 | | 25.0 | 32.0 | 7.0 | 27.9 | 1.9 | 6.9 |  | **0.03** |
| From bloom to harvest (days) | 200 | 65.0 | 71.0 | 6.0 | 65.5 | 1.3 | 2.0 |  | 36 | | 65.0 | 71.0 | 6.0 | 65.9 | 1.7 | 2.6 |  | 0.09 |
| Pod length (cm) | 200 | 1.3 | 5.8 | 4.5 | 4.6 | 0.5 | 10.9 |  | 36 | | 1.3 | 5.5 | 4.2 | 4.4 | 0.8 | 18.2 |  | 0.15 |
| Pod width (cm) | 200 | 0.9 | 4.3 | 3.4 | 1.2 | 0.2 | 20.0 |  | 36 | | 0.9 | 4.3 | 3.4 | 1.3 | 0.5 | 41.9 |  | 0.30 |
| Single pod weight (g) | 200 | 0.9 | 3.3 | 2.4 | 2.1 | 0.4 | 20.5 |  | 36 | | 0.9 | 3.1 | 2.2 | 2.1 | 0.5 | 26.1 |  | 0.27 |
| Number of pods per 500g | 200 | 140.0 | 282.0 | 142.0 | 186.8 | 33.2 | 17.8 |  | 36 | | 140.0 | 282.0 | 142.0 | 198.8 | 42.1 | 21.2 |  | 0.11 |
| First pod height (cm) | 200 | 4.2 | 22.3 | 18.1 | 11.5 | 4.3 | 37.3 |  | 36 | | 4.3 | 21.0 | 16.7 | 12.3 | 4.7 | 38.0 |  | 0.34 |
| Number of seeds per pod | 200 | 2.0 | 4.0 | 2.0 | 3.0 | 0.9 | 29.9 |  | 36 | | 2.0 | 4.0 | 2.0 | 2.9 | 0.9 | 30.3 |  | 1.00 |
| Shelling rate (%) | 200 | 4.8 | 75.0 | 70.2 | 57.2 | 7.6 | 13.2 |  | 36 | | 4.8 | 75.0 | 70.2 | 56.6 | 10.9 | 19.3 |  | 0.70 |
| Immature seed length (mm) | 200 | 6.0 | 18.1 | 12.1 | 15.5 | 1.6 | 10.0 |  | 36 | | 6.0 | 18.1 | 12.1 | 15.0 | 2.3 | 15.5 |  | 0.10 |
| Immature seed width (mm) | 200 | 1.1 | 40.9 | 39.8 | 11.0 | 2.8 | 25.6 |  | 36 | | 1.1 | 40.9 | 39.8 | 11.3 | 5.5 | 48.9 |  | 0.63 |
| Immature seed thickness (mm) | 200 | 5.8 | 9.7 | 3.9 | 8.1 | 0.8 | 10.4 |  | 36 | | 6.0 | 9.7 | 3.7 | 8.0 | 0.9 | 10.6 |  | 0.83 |
| 100 immature seed weight (g) | 200 | 6.2 | 100.0 | 93.8 | 68.7 | 14.6 | 21.3 |  | 36 | | 6.8 | 100.0 | 93.2 | 62.4 | 18.6 | 29.9 |  | **0.02** |
| Abbreviation: N, number of germplasms; SD, standard deviation; CV, coefficient of variation; *p*-value, *p*-value of difference test (Student’s *t*-test or Welch’s *t*-test).  ^a^CC_impu_ was selected from 200 accessions and 46 complete (observed plus imputed values) phenotypic traits by using PowerCore. ^b^Student’s *t*-test (if assumption of homogeneity of variance is met) and Welch’s *t*-test (if assumption of homogeneity of variance is not met) were used to conduct mean difference among two collections (*p*-value smaller than 0.05 is highlighted in bold). | | | | | | | | | | | | | | | | | | |

**Supplementary Table 7.** Difference tests of qualitative traits between the core collection and the complete entire collection in Taiwanese vegetable soybean germplasms.

| **Phenotypic trait** | **Complete entire collection (EC_impu_)** | |  | **Core collection^a^ (CC_impu_)** | |  | **Difference test**  **(*p*-value)^b^** |
| --- | --- | --- | --- | --- | --- | --- | --- |
|  | **N** | **(%)** |  | **N** | **(%)** |  |  |
| Seed shape |  |  |  |  |  |  | 0.65 |
| Round | 107 | (53.5) |  | 16 | (44.4) |  |  |
| Oblate | 21 | (10.5) |  | 6 | (16.7) |  |  |
| Oval | 59 | (29.5) |  | 11 | (30.6) |  |  |
| Flat | 13 | (6.5) |  | 3 | (8.3) |  |  |
| Seed coat color |  |  |  |  |  |  | 0.33 |
| Yellowish white | 26 | (13.0) |  | 4 | (11.2) |  |  |
| Yellow | 71 | (35.5) |  | 15 | (41.7) |  |  |
| Green | 101 | (50.5) |  | 15 | (41.7) |  |  |
| Pale brown | 1 | (0.5) |  | 1 | (2.7) |  |  |
| Reddish brown | 1 | (0.5) |  | 1 | (2.7) |  |  |
| Hilum color |  |  |  |  |  |  | 0.40 |
| Light yellow | 15 | (7.5) |  | 5 | (13.9) |  |  |
| Yellow | 62 | (31.0) |  | 12 | (33.3) |  |  |
| Brown | 118 | (59.0) |  | 17 | (47.2) |  |  |
| Green | 5 | (2.5) |  | 2 | (5.6) |  |  |
| Hypocotyl coloration |  |  |  |  |  |  | 0.18 |
| Green | 132 | (66.0) |  | 19 | (52.8) |  |  |
| Purple | 68 | (34.0) |  | 17 | (47.2) |  |  |
| Number of nodes on main stem |  |  |  |  |  |  | 0.52 |
| Medium | 147 | (73.5) |  | 24 | (66.7) |  |  |
| Large | 53 | (26.5) |  | 12 | (33.3) |  |  |
| Stem color |  |  |  |  |  |  | 0.73 |
| Light green | 106 | (53.0) |  | 17 | (47.2) |  |  |
| Green | 75 | (37.5) |  | 16 | (44.5) |  |  |
| Dark green | 19 | (9.5) |  | 3 | (8.3) |  |  |
| Number of branches |  |  |  |  |  |  | 0.68 |
| Low | 59 | (29.5) |  | 9 | (25.0) |  |  |
| Medium | 79 | (39.5) |  | 17 | (47.2) |  |  |
| High | 62 | (31.0) |  | 10 | (27.8) |  |  |
| Lodging score |  |  |  |  |  |  | 0.13 |
| Absent | 116 | (58.0) |  | 23 | (63.9) |  |  |
| Medium | 51 | (25.5) |  | 4 | (11.1) |  |  |
| High | 33 | (16.5) |  | 9 | (25.0) |  |  |
| Leaflet size |  |  |  |  |  |  | 0.86 |
| Small | 92 | (46.0) |  | 23 | (63.9) |  |  |
| Medium | 85 | (42.5) |  | 4 | (11.1) |  |  |
| Large | 23 | (11.5) |  | 9 | (25.0) |  |  |
| Leaflet shape |  |  |  |  |  |  | 0.58 |
| Lanceolate | 1 | (0.5) |  | 1 | (2.8) |  |  |
| Lanceolate to oblong | 54 | (27.0) |  | 9 | (25.0) |  |  |
| Rhomboid | 30 | (15.0) |  | 4 | (11.1) |  |  |
| Oval | 62 | (31.0) |  | 10 | (27.8) |  |  |
| Elliptic | 53 | (26.5) |  | 12 | (33.3) |  |  |
| Leaf color |  |  |  |  |  |  | 0.40 |
| Green | 55 | (27.5) |  | 13 | (36.1) |  |  |
| Dark green | 145 | (72.5) |  | 23 | (63.9) |  |  |
| Plant type |  |  |  |  |  |  | 0.29 |
| Determinate | 163 | (81.5) |  | 26 | (72.2) |  |  |
| Semi-determinate | 37 | (18.5) |  | 10 | (27.8) |  |  |
| Pubescence density |  |  |  |  |  |  | 0.49 |
| Absent | 5 | (2.5) |  | 2 | (5.6) |  |  |
| Rare | 20 | (10.0) |  | 6 | (16.7) |  |  |
| Sparse | 33 | (16.5) |  | 7 | (19.4) |  |  |
| Medium | 86 | (43.0) |  | 11 | (30.5) |  |  |
| Dense | 56 | (28.0) |  | 10 | (27.8) |  |  |
| Pubescence color |  |  |  |  |  |  | 0.43 |
| Greyish white | 103 | (51.5) |  | 15 | (41.7) |  |  |
| Pale brown | 63 | (31.5) |  | 12 | (33.3) |  |  |
| Brown | 34 | (17.0) |  | 9 | (25.0) |  |  |
| Corolla color |  |  |  |  |  |  | 0.25 |
| White | 138 | (69.0) |  | 20 | (55.6) |  |  |
| Purple throat | 35 | (17.5) |  | 8 | (22.2) |  |  |
| Purple | 27 | (13.5) |  | 8 | (22.2) |  |  |
| Pod set capacity |  |  |  |  |  |  | 0.99 |
| Low | 30 | (15.0) |  | 5 | (13.9) |  |  |
| Medium | 77 | (38.5) |  | 13 | (36.1) |  |  |
| High | 93 | (46.5) |  | 18 | (50.0) |  |  |
| Pod length |  |  |  |  |  |  | **0.04** |
| Short | 44 | (22.0) |  | 14 | (38.9) |  |  |
| Medium | 110 | (55.0) |  | 12 | (33.3) |  |  |
| Long | 46 | (23.0) |  | 10 | (27.8) |  |  |
| Pod width |  |  |  |  |  |  | 0.37 |
| Narrow | 58 | (29.0) |  | 13 | (36.1) |  |  |
| Medium | 87 | (43.5) |  | 17 | (47.2) |  |  |
| Broad | 55 | (27.5) |  | 6 | (16.7) |  |  |
| Pod shape |  |  |  |  |  |  | 1.00 |
| Sword shaped | 147 | (92.0) |  | 26 | (72.2) |  |  |
| Sickle shaped | 53 | (8.0) |  | 10 | (27.8) |  |  |
| Pod color |  |  |  |  |  |  | 0.49 |
| Light green | 49 | (24.5) |  | 12 | (33.3) |  |  |
| Green | 125 | (62.5) |  | 19 | (52.8) |  |  |
| Dark green | 26 | (13.0) |  | 5 | (13.9) |  |  |
| Immature seed size |  |  |  |  |  |  | 0.85 |
| Small | 47 | (23.5) |  | 10 | (27.8) |  |  |
| Medium | 102 | (51.0) |  | 17 | (47.2) |  |  |
| Large | 51 | (25.5) |  | 9 | (25.0) |  |  |
| Immature seed coat color |  |  |  |  |  |  | 0.05 |
| Light green | 14 | (7.0) |  | 6 | (16.7) |  |  |
| Green | 158 | (79.0) |  | 22 | (61.1) |  |  |
| Purple | 28 | (14.0) |  | 8 | (22.2) |  |  |
| Immature seed texture |  |  |  |  |  |  | 1.00 |
| Soft | 75 | (37.5) |  | 14 | (38.9) |  |  |
| Slightly soft | 125 | (62.5) |  | 22 | (61.1) |  |  |
| Easiness of pod removal |  |  |  |  |  |  | 0.90 |
| High | 98 | (49.0) |  | 19 | (52.8) |  |  |
| Medium | 22 | (11.0) |  | 4 | (11.1) |  |  |
| Low | 80 | (40.0) |  | 13 | (36.1) |  |  |
| Storability |  |  |  |  |  |  | 0.87 |
| Poor | 46 | (23.0) |  | 46 | (19.4) |  |  |
| Medium | 71 | (35.5) |  | 71 | (38.9) |  |  |
| Good | 83 | (41.5) |  | 83 | (41.7) |  |  |
| Abbreviation: N, number of germplasms; %, the percentage of each types accounted for the trait.  ^a^CC_impu_ was selected from 200 accessions and 46 complete (observed plus imputed values) phenotypes by using PowerCore. ^b^Chi-squared test was conducted to test difference among the EC_impu_ and the CC_impu_ (*p*-value smaller than 0.05 is highlighted in bold). | | | | | | | |

**Supplementary Table 8.** Difference tests of quantitative traits (observed values only) between the core collection and the entire collection in Taiwanese vegetable soybean germplasms.

| **Phenotypic trait** | **Observed entire collection (EC_raw_)** | | | | | | |  | | **Core collection (CC_raw_)^a^** | | | | | | |  | **Difference test^b^** |
| --- | --- | --- | --- | --- | --- | --- | --- | --- | --- | --- | --- | --- | --- | --- | --- | --- | --- | --- |
|  | **N** | **Min** | **Max** | **Range** | **Mean** | **SD** | **CV(%)** |  | **N** | | **Min** | **Max** | **Range** | **Mean** | **SD** | **CV(%)** |  | ***p*-value** |
| Seed length (mm) | 200 | 7.1 | 11.2 | 4.1 | 9.0 | 0.8 | 8.7 |  | 43 | | 7.1 | 11.1 | 4.0 | 9.0 | 1.0 | 11.6 |  | 0.68 |
| Seed width (mm) | 200 | 6.2 | 9.7 | 3.5 | 8.3 | 0.6 | 7.2 |  | 43 | | 6.2 | 9.7 | 3.5 | 8.1 | 0.8 | 9.6 |  | 0.21 |
| Seed thickness (mm) | 200 | 5.1 | 8.8 | 3.7 | 7.1 | 0.7 | 9.4 |  | 43 | | 5.1 | 8.8 | 3.7 | 6.9 | 0.8 | 11.3 |  | 0.06 |
| 100 seed weight (g) | 200 | 4.2 | 51.2 | 47.0 | 33.4 | 7.5 | 22.5 |  | 43 | | 4.2 | 51.2 | 47.0 | 30.9 | 10.3 | 33.5 |  | 0.14 |
| Internode length (cm) | 50 | 1.7 | 7.0 | 5.3 | 3.8 | 1.0 | 26.1 |  | 9 | | 2.2 | 7.0 | 4.8 | 4.0 | 1.6 | 39.2 |  | 0.65 |
| Plant height (cm) | 198 | 17.3 | 70.7 | 53.4 | 37.1 | 10.7 | 28.9 |  | 43 | | 17.3 | 70.7 | 53.4 | 39.1 | 12.5 | 32.0 |  | 0.29 |
| Leaflet length (cm) | 200 | 7.0 | 116.0 | 109.0 | 10.6 | 7.6 | 71.8 |  | 43 | | 7.4 | 116.0 | 108.6 | 13.0 | 16.2 | 124.5 |  | 0.14 |
| Leaflet width (cm) | 200 | 1.4 | 10.4 | 9.0 | 7.2 | 1.2 | 16.4 |  | 43 | | 2.2 | 10.4 | 8.2 | 7.11 | 1.4 | 20.2 |  | 0.60 |
| From sowing to flowering (days) | 49 | 25.0 | 32.0 | 7.0 | 27.5 | 1.8 | 6.4 |  | 12 | | 25.0 | 32.0 | 7.0 | 27.8 | 2.2 | 7.8 |  | 0.52 |
| From bloom to harvest (days) | 43 | 65.0 | 71.0 | 6.0 | 66.4 | 1.7 | 2.6 |  | 11 | | 65.0 | 70.0 | 5.0 | 66.8 | 1.7 | 2.5 |  | 0.45 |
| Pod length (cm) | 149 | 1.3 | 5.8 | 4.5 | 4.6 | 0.5 | 11.6 |  | 34 | | 1.3 | 5.8 | 4.5 | 4.5 | 0.8 | 18.0 |  | 0.25 |
| Pod width (cm) | 149 | 0.9 | 4.3 | 3.4 | 1.2 | 0.3 | 22.6 |  | 34 | | 0.9 | 4.3 | 3.4 | 1.3 | 0.6 | 43.0 |  | 0.44 |
| Single pod weight (g) | 99 | 0.9 | 3.3 | 2.4 | 2.1 | 0.5 | 23.3 |  | 25 | | 0.9 | 3.3 | 2.4 | 2.1 | 0.6 | 29.0 |  | 0.93 |
| Number of pods per 500g | 99 | 140.0 | 282.0 | 142.0 | 187.8 | 33.5 | 17.8 |  | 25 | | 140.0 | 265.0 | 125.0 | 196.5 | 39.7 | 20.2 |  | 0.27 |
| First pod height (cm) | 150 | 4.2 | 22.3 | 18.1 | 11.9 | 4.4 | 37.3 |  | 34 | | 4.2 | 21.0 | 16.8 | 11.6 | 5.1 | 43.8 |  | 0.76 |
| Number of seeds per pod | 50 | 2.0 | 4.0 | 2.0 | 2.7 | 0.9 | 32.0 |  | 9 | | 2.0 | 4.0 | 2.0 | 2.9 | 0.9 | 32.1 |  | 0.55 |
| Shelling rate (%) | 150 | 4.8 | 75.0 | 70.2 | 56.8 | 7.0 | 12.3 |  | 34 | | 4.8 | 75.0 | 70.2 | 55.1 | 10.6 | 19.2 |  | 0.25 |
| Immature seed length (mm) | 149 | 6.0 | 18.1 | 12.1 | 15.5 | 1.6 | 10.5 |  | 33 | | 6.0 | 18.1 | 12.1 | 15.2 | 2.4 | 15.5 |  | 0.41 |
| Immature seed width (mm) | 150 | 1.1 | 40.9 | 39.8 | 11.0 | 3.1 | 28.3 |  | 34 | | 1.1 | 40.9 | 39.8 | 11.3 | 5.8 | 50.9 |  | 0.68 |
| Immature seed thickness (mm) | 150 | 5.8 | 9.7 | 3.9 | 8.1 | 0.8 | 10.2 |  | 34 | | 6.0 | 9.7 | 3.7 | 7.9 | 0.9 | 10.9 |  | 0.33 |
| 100 immature seed weight (g) | 149 | 6.2 | 100.0 | 93.8 | 69.1 | 14.9 | 21.5 |  | 34 | | 6.8 | 98.0 | 91.2 | 63.1 | 18.6 | 29.5 |  | 0.05 |
| Abbreviation: N, number of germplasms; SD, standard deviation; CV, coefficient of variation; *p*-value, *p*-value of difference test (Student’s *t*-test or Welch’s *t*-test).  ^a^CC_raw_ was selected from 200 accessions and 46 phenotypes (observed values only) by using PowerCore. ^b^Student’s *t*-test (if assumption of homogeneity of variance is met) and Welch’s *t*-test (if assumption of homogeneity of variance is not met) were used to conduct mean difference among two collections. | | | | | | | | | | | | | | | | | | |

**Supplementary Table 9.** Difference tests of qualitative traits (observed values only) between the core collection and the entire collection in Taiwanese vegetable soybean germplasms.

| **Phenotypic trait** | **Observed entire collection (EC_raw_)** | |  | **Core collection (CC_raw_)^a^** | |  | **Difference test^b^** |
| --- | --- | --- | --- | --- | --- | --- | --- |
|  | **N** | **Classes** |  | **N** | **Classes** |  | ***p*-value** |
| Seed shape | 199 | 4 |  | 43 | 4 |  | 0.27 |
| Seed coat color | 189 | 5 |  | 40 | 5 |  | 0.32 |
| Hilum color | 177 | 4 |  | 35 | 4 |  | 0.64 |
| Hypocotyl coloration | 199 | 2 |  | 43 | 2 |  | 0.29 |
| Number of nodes on main stem | 99 | 2 |  | 25 | 2 |  | 0.95 |
| Stem color | 182 | 3 |  | 39 | 3 |  | 0.71 |
| Number of branches | 199 | 3 |  | 43 | 3 |  | 0.73 |
| Lodging score | 71 | 3 |  | 13 | 3 |  | 0.27 |
| Leaflet size | 150 | 3 |  | 30 | 3 |  | 0.69 |
| Leaflet shape | 147 | 5 |  | 33 | 5 |  | 0.50 |
| Leaf color | 150 | 2 |  | 34 | 2 |  | 0.33 |
| Plant type | 149 | 2 |  | 33 | 2 |  | 0.56 |
| Pubescence density | 199 | 5 |  | 43 | 5 |  | 0.46 |
| Pubescence color | 197 | 3 |  | 42 | 3 |  | 0.30 |
| Corolla color | 199 | 3 |  | 43 | 3 |  | 0.51 |
| Pod set capacity | 149 | 3 |  | 34 | 3 |  | 0.91 |
| Pod length | 95 | 3 |  | 25 | 3 |  | 0.41 |
| Pod width | 98 | 3 |  | 25 | 3 |  | 0.94 |
| Pod shape | 99 | 2 |  | 25 | 2 |  | 0.17 |
| Pod color | 48 | 3 |  | 9 | 3 |  | 0.59 |
| Immature seed size | 99 | 3 |  | 22 | 3 |  | 0.66 |
| Immature seed coat color | 99 | 3 |  | 22 | 3 |  | 0.57 |
| Immature seed texture | 50 | 2 |  | 9 | 2 |  | 1.00 |
| Easiness of pod removal | 50 | 3 |  | 9 | 3 |  | 0.91 |
| Storability | 50 | 3 |  | 9 | 3 |  | 0.86 |
| Abbreviation: N, number of germplasms; Classes, types of the traits.  ^a^CC_raw_ was selected from 200 accessions and 46 phenotypes (observed values only) by using PowerCore. ^b^Chi-squared test was conducted to test for difference among two collections. | | | | | | | |

**Supplementary Table 10.** Diversity comparisons between the core collection and the complete entire collection in Taiwanese vegetable soybean germplasms.

| Phenotypic traits | Clusters & diversity | | | | | | |  | Diversity  retained / lost | |
| --- | --- | --- | --- | --- | --- | --- | --- | --- | --- | --- |
|  | Complete  entire collection  (EC_impu_) | | |  | Core collection  (CC_impu_)^a^ | | |  |  |  |
|  | *k*_EC_^b^ | H′ | Nei’s |  | *k*_CC_ | H′ | Nei’s |  | H′ | Nei’s |
| **Quantitative traits** |  |  |  |  |  |  |  |  |  |  |
| Seed length (mm) | 7 | 0.93 | 0.82 |  | 7 | 0.92 | 0.82 |  | -2% | **0%** |
| Seed width (mm) | 7 | 0.91 | 0.81 |  | 7 | 0.97 | 0.84 |  | **7%** | **4%** |
| Seed thickness (mm) | 7 | 0.98 | 0.85 |  | 7 | 0.96 | 0.83 |  | -3% | -2% |
| 100 seed weight (g) | 7 | 0.99 | 0.85 |  | 7 | 0.97 | 0.84 |  | -2% | -1% |
| Internode length (cm) | 7 | 0.96 | 0.84 |  | 7 | 0.99 | 0.85 |  | **3%** | **2%** |
| Plant height (cm) | 6 | 0.98 | 0.82 |  | 6 | 0.85 | 0.74 |  | -13% | -10% |
| Leaflet length (cm) | 8 | 0.92 | 0.84 |  | 8 | 0.87 | 0.80 |  | -5% | -6% |
| Leaflet width (cm) | 8 | 0.91 | 0.84 |  | 8 | 0.94 | 0.85 |  | **2%** | **1%** |
| From sowing to flowering (days) | 4 | 0.93 | 0.71 |  | 4 | 0.94 | 0.71 |  | **1%** | **0%** |
| From bloom to harvest (days) | 2 | 0.33 | 0.11 |  | 2 | 0.58 | 0.24 |  | **78%** | **112%** |
| Pod length (cm) | 7 | 0.92 | 0.82 |  | 7 | 0.85 | 0.77 |  | -7% | -7% |
| Pod width (cm) | 3 | 0.99 | 0.66 |  | 3 | 0.99 | 0.66 |  | **0%** | **0%** |
| Single pod weight (g) | 8 | 0.90 | 0.82 |  | 7 | 0.93 | 0.83 |  | **4%** | **0%** |
| Number of pods per 500g | 7 | 0.92 | 0.82 |  | 7 | 0.92 | 0.82 |  | **0%** | -1% |
| First pod height (cm) | 6 | 0.98 | 0.82 |  | 6 | 0.95 | 0.80 |  | -3% | -2% |
| Number of seeds per pod | 3 | 0.97 | 0.65 |  | 3 | 0.97 | 0.65 |  | **0%** | **0%** |
| Shelling rate (%) | 10 | 0.92 | 0.86 |  | 10 | 0.88 | 0.83 |  | -4% | -4% |
| Immature seed length (mm) | 7 | 0.91 | 0.81 |  | 7 | 0.96 | 0.83 |  | **5%** | **3%** |
| Immature seed width (mm) | 8 | 0.90 | 0.84 |  | 8 | 0.91 | 0.82 |  | **0%** | -1% |
| Immature seed thickness (mm) | 7 | 0.95 | 0.83 |  | 7 | 0.89 | 0.79 |  | -6% | -4% |
| 100 immature seed weight (g) | 8 | 0.92 | 0.84 |  | 8 | 0.80 | 0.74 |  | -13% | -11% |
| **Qualitative traits** |  |  |  |  |  |  |  |  |  |  |
| Seed shape | 4 | 0.80 | 0.61 |  | 4 | 0.89 | 0.67 |  | **11%** | **10%** |
| Seed coat color | 5 | 0.64 | 0.60 |  | 5 | 0.73 | 0.64 |  | **14%** | **7%** |
| Hilum color | 4 | 0.69 | 0.55 |  | 4 | 0.83 | 0.64 |  | **20%** | **16%** |
| Hypocotyl coloration | 2 | 0.92 | 0.45 |  | 2 | 1.00 | 0.50 |  | **9%** | **11%** |
| Number of nodes on main stem | 2 | 0.83 | 0.39 |  | 2 | 0.92 | 0.44 |  | **11%** | **13%** |
| Stem color | 3 | 0.84 | 0.57 |  | 3 | 0.84 | 0.57 |  | **0%** | **0%** |
| Number of branches | 3 | 0.99 | 0.66 |  | 3 | 0.96 | 0.64 |  | -3% | -3% |
| Lodging score | 3 | 0.88 | 0.57 |  | 3 | 0.80 | 0.52 |  | -9% | -9% |
| Leaflet size | 3 | 0.88 | 0.59 |  | 3 | 0.91 | 0.61 |  | **3%** | **3%** |
| Leaflet shape | 5 | 0.86 | 0.74 |  | 5 | 0.88 | 0.74 |  | **2%** | **0%** |
| Leaf color | 2 | 0.85 | 0.40 |  | 2 | 0.94 | 0.46 |  | **11%** | **15%** |
| Plant type | 2 | 0.69 | 0.30 |  | 2 | 0.85 | 0.40 |  | **23%** | **33%** |
| Pubescence density | 5 | 0.83 | 0.70 |  | 5 | 0.93 | 0.76 |  | **12%** | **9%** |
| Pubescence color | 3 | 0.92 | 0.61 |  | 3 | 0.98 | 0.65 |  | **7%** | **7%** |
| Corolla color | 3 | 0.76 | 0.48 |  | 3 | 0.91 | 0.59 |  | **20%** | **23%** |
| Pod set capacity | 3 | 0.92 | 0.61 |  | 3 | 0.90 | 0.60 |  | -2% | -2% |
| Pod length | 3 | 0.91 | 0.60 |  | 3 | 0.99 | 0.66 |  | **9%** | **10%** |
| Pod width | 3 | 0.98 | 0.65 |  | 3 | 0.93 | 0.62 |  | -5% | -5% |
| Pod shape | 2 | 0.83 | 0.39 |  | 2 | 0.85 | 0.40 |  | **2%** | **3%** |
| Pod color | 3 | 0.82 | 0.53 |  | 3 | 0.89 | 0.59 |  | **9%** | **11%** |
| Immature seed size | 3 | 0.94 | 0.62 |  | 3 | 0.96 | 0.64 |  | **2%** | **3%** |
| Immature seed coat color | 3 | 0.59 | 0.35 |  | 3 | 0.85 | 0.55 |  | **44%** | **57%** |
| Immature seed texture | 2 | 0.95 | 0.47 |  | 2 | 0.96 | 0.48 |  | **1%** | **2%** |
| Easiness of pod removal | 3 | 0.87 | 0.59 |  | 3 | 0.86 | 0.58 |  | -1% | -2% |
| Storability | 3 | 0.97 | 0.65 |  | 3 | 0.96 | 0.64 |  | -1% | -2% |
| Abbreviation: *k*_EC_, number of clusters in the EC_impu_; *k*_CC_, number of clusters in the CC_impu_; H′, Shannon-Weaver diversity index; Nei’s, Nei’s diversity index.  ^a^CC_impu_ was selected using PowerCore for mixed-type phenotypic traits. ^b^Clustering analyses for quantitative traits were conducted using weighted *k*-means clustering algorithm. Phenotypic diversity richness or evenness retained in the CC_impu_ is highlighted in bold. | | | | | | | | | | |

**Supplementary Table 11.** Diversity comparisons between the core collection and the observed entire collection in vegetable soybean in Taiwanese vegetable soybean germplasms.

| Phenotypic traits | Clusters & diversity | | | | | | | | |  | Diversity  retained / lost | |
| --- | --- | --- | --- | --- | --- | --- | --- | --- | --- | --- | --- | --- |
|  | Observed  entire collection  (EC_raw_) | | | |  | Core collection  (CC_raw_)^a^ | | | |  |  |  |
|  | N | *k*_EC_^b^ | H′ | Nei’s |  | N | *k*_CC_ | H′ | Nei’s |  | H′ | Nei’s |
| **Quantitative traits** |  |  |  |  |  |  |  |  |  |  |  |  |
| Seed length (mm) | 200 | 6 | 0.95 | 0.81 |  | 43 | 6 | 0.99 | 0.83 |  | **4%** | **2%** |
| Seed width (mm) | 200 | 7 | 0.96 | 0.84 |  | 43 | 7 | 0.92 | 0.82 |  | **3%** | **1%** |
| Seed thickness (mm) | 200 | 6 | 0.97 | 0.81 |  | 43 | 6 | 0.96 | 0.81 |  | -2% | -1% |
| 100 seed weight (g) | 200 | 6 | 0.97 | 0.81 |  | 43 | 6 | 0.96 | 0.81 |  | **3%** | **2%** |
| Internode length (cm) | 50 | 7 | 0.92 | 0.81 |  | 9 | 5 | 0.73 | 0.72 |  | -19% | -10% |
| Plant height (cm) | 198 | 6 | 0.95 | 0.80 |  | 43 | 6 | 0.94 | 0.80 |  | -3% | -2% |
| Leaflet length (cm) | 200 | 8 | 0.90 | 0.83 |  | 43 | 8 | 0.95 | 0.85 |  | **3%** | **1%** |
| Leaflet width (cm) | 200 | 8 | 0.90 | 0.83 |  | 43 | 8 | 0.93 | 0.84 |  | **2%** | **0%** |
| From sowing to flowering (days) | 49 | 6 | 0.79 | 0.71 |  | 12 | 6 | 0.95 | 0.81 |  | **13%** | **8%** |
| From bloom to harvest (days) | 43 | 6 | 0.36 | 0.35 |  | 11 | 2 | 0.99 | 0.50 |  | **3%** | **4%** |
| Pod length (cm) | 149 | 9 | 0.93 | 0.86 |  | 34 | 9 | 0.96 | 0.87 |  | **2%** | **1%** |
| Pod width (cm) | 149 | 2 | 0.90 | 0.44 |  | 34 | 2 | 0.87 | 0.42 |  | -3% | -4% |
| Single pod weight (g) | 99 | 10 | 0.94 | 0.87 |  | 25 | 10 | 0.90 | 0.85 |  | **3%** | **1%** |
| Number of pods per 500g | 99 | 7 | 0.96 | 0.84 |  | 25 | 7 | 0.94 | 0.83 |  | -1% | **1%** |
| First pod height (cm) | 150 | 6 | 0.98 | 0.82 |  | 34 | 6 | 0.98 | 0.82 |  | **0%** | **0%** |
| Number of seeds per pod | 50 | 2 | 0.98 | 0.49 |  | 9 | 2 | 0.99 | 0.49 |  | **0%** | **0%** |
| Shelling rate (%) | 150 | 9 | 0.91 | 0.85 |  | 33 | 9 | 0.91 | 0.84 |  | **1%** | **0%** |
| Immature seed length (mm) | 149 | 7 | 0.92 | 0.82 |  | 32 | 7 | 0.88 | 0.78 |  | -3% | -5% |
| Immature seed width (mm) | 150 | 7 | 0.95 | 0.83 |  | 33 | 7 | 0.92 | 0.82 |  | **4%** | **2%** |
| Immature seed thickness (mm) | 150 | 7 | 0.98 | 0.84 |  | 33 | 7 | 0.93 | 0.81 |  | -3% | -2% |
| 100 immature seed weight (g) | 149 | 9 | 0.96 | 0.87 |  | 33 | 9 | 0.89 | 0.83 |  | **4%** | **2%** |
| **Qualitative traits** |  |  |  |  |  |  |  |  |  |  |  |  |
| Seed shape | 199 | 4 | 0.80 | 0.61 |  | 43 | 4 | 0.87 | 0.67 |  | **9%** | **9%** |
| Seed coat color | 189 | 5 | 0.65 | 0.61 |  | 40 | 5 | 0.77 | 0.68 |  | **19%** | **11%** |
| Hilum color | 177 | 4 | 0.69 | 0.55 |  | 35 | 4 | 0.75 | 0.59 |  | **8%** | **7%** |
| Hypocotyl coloration | 199 | 2 | 0.93 | 0.45 |  | 43 | 2 | 0.99 | 0.49 |  | **7%** | **10%** |
| Number of nodes on main stem | 99 | 2 | 0.44 | 0.17 |  | 25 | 2 | 0.53 | 0.21 |  | **20%** | **28%** |
| Stem color | 182 | 3 | 0.75 | 0.52 |  | 39 | 3 | 0.72 | 0.52 |  | -4% | **0%** |
| Number of branches | 199 | 3 | 0.99 | 0.66 |  | 43 | 3 | 0.97 | 0.65 |  | -2% | -2% |
| Lodging score | 71 | 3 | 0.33 | 0.18 |  | 13 | 3 | 0.63 | 0.38 |  | **90%** | **110%** |
| Leaflet size | 150 | 3 | 0.81 | 0.55 |  | 30 | 3 | 0.74 | 0.53 |  | -9% | -5% |
| Leaflet shape | 147 | 5 | 0.87 | 0.74 |  | 33 | 5 | 0.88 | 0.73 |  | **2%** | -1% |
| Leaf color | 150 | 2 | 0.89 | 0.43 |  | 34 | 2 | 0.98 | 0.48 |  | **10%** | **14%** |
| Plant type | 149 | 2 | 0.27 | 0.09 |  | 33 | 2 | 0.44 | 0.17 |  | **61%** | **85%** |
| Pubescence density | 199 | 5 | 0.82 | 0.70 |  | 43 | 5 | 0.87 | 0.73 |  | **6%** | **5%** |
| Pubescence color | 197 | 3 | 0.91 | 0.60 |  | 42 | 3 | 0.96 | 0.64 |  | **5%** | **5%** |
| Corolla color | 199 | 3 | 0.75 | 0.47 |  | 43 | 3 | 0.85 | 0.55 |  | **14%** | **17%** |
| Pod set capacity | 149 | 3 | 0.83 | 0.56 |  | 34 | 3 | 0.81 | 0.54 |  | -2% | -3% |
| Pod length | 95 | 3 | 0.87 | 0.57 |  | 25 | 3 | 0.97 | 0.65 |  | **12%** | **14%** |
| Pod width | 98 | 3 | 0.97 | 0.64 |  | 25 | 3 | 0.96 | 0.63 |  | -1% | -2% |
| Pod shape | 99 | 2 | 0.41 | 0.15 |  | 25 | 2 | 0.72 | 0.32 |  | **78%** | **115%** |
| Pod color | 48 | 3 | 0.37 | 0.19 |  | 9 | 3 | 0.62 | 0.37 |  | **69%** | **93%** |
| Immature seed size | 99 | 3 | 0.91 | 0.60 |  | 22 | 3 | 0.96 | 0.64 |  | **6%** | **7%** |
| Immature seed coat color | 99 | 3 | 0.38 | 0.20 |  | 22 | 3 | 0.55 | 0.31 |  | **42%** | **55%** |
| Immature seed texture | 50 | 2 | 0.33 | 0.11 |  | 9 | 2 | 0.50 | 0.20 |  | **54%** | **75%** |
| Easiness of pod removal | 50 | 3 | 0.77 | 0.51 |  | 9 | 3 | 0.77 | 0.49 |  | **1%** | -2% |
| Storability | 50 | 3 | 0.94 | 0.62 |  | 9 | 3 | 0.85 | 0.57 |  | -9% | -9% |
| Abbreviation: N, number of germplasms; *k*_EC_, number of clusters in the EC_raw_; *k*_CC_, number of clusters in the CC_raw_; H′, Shannon-Weaver diversity index; Nei’s, Nei’s diversity index.  ^a^CC_raw_ was identified using PowerCore for mixed-type phenotypic traits. ^b^Clustering analyses for quantitative traits were conducted using weighted *k*-means clustering algorithm. Phenotypic diversity richness or evenness retained in the CC_raw_ is highlighted in bold. | | | | | | | | | | | | |

**Supplementary Table 12.** Evaluation in percentage of the trait differences between the CC and the EC of three subsets in vegetable soybean.

| **Subset** | **N** | **Property** | | | | | | | | | | | | | |
| --- | --- | --- | --- | --- | --- | --- | --- | --- | --- | --- | --- | --- | --- | --- | --- |
|  |  | **MD%**^a^ | |  | **VD%**^a^ | |  | **CR%**^a^ | |  | **VR%**^a^ | |  | **Coverage**^b^ | |
|  |  | Observed | Complete |  | Observed | Complete |  | Observed | Complete |  | Observed | Complete |  | Observed | Complete |
| CC_raw_∩CC_impu_ | 21 | 6.16 | 5.80 |  | 46.51 | 45.65 |  | 88.90 | 90.77 |  | 153.39 | 147.52 |  | 86.53 | 97.87 |
| CC_raw_\CC_impu_ | 22 | 3.27 | 2.13 |  | 172.44 | 169.14 |  | 66.20 | 67.80 |  | 105.45 | 100.37 |  | 94.93 | 97.60 |
| CC_impu_\CC_raw_ | 15 | 4.33 | 3.40 |  | NA | 222.15 |  | 55.50 | 69.94 |  | NA | 112.88 |  | 81.27 | 98.40 |
| Abbreviation: CC_raw_ and CC_impu_, CC_raw_ and CC_impu_ was identified by PowerCore with observed and complete phenotypes, respectively; CC_raw_∩CC_impu_, intersection of CC_raw_ and CC_impu_; CC_raw_\CC_impu_, difference of CC_raw_ from CC_impu_; CC_impu_\CC_raw_, difference of CC_impu_ from CC_raw_; N, number of subset; MD%, mean difference percentage; VD%, variance difference percentage; CR%, coincidence rate; VR%, variable rate; Observed, computed the properties of three subsets based on EC_raw_ and CC_raw_; Complete, computed the properties of three subsets based on EC_impu_ and CC_impu_;.  ^a^MD%, VD%, CR% and VR% were computed using all 25 quantitative traits for observed and complete data (observed plus imputed values), respectively. ^b^Coverages were computed using all 21 qualitative traits by observed and complete data, respectively. | | | | | | | | | | | | | | | |

**Supplementary Figure 1.** The Venn diagram of the three sets of core collection (CC_raw_∩CC_impu_, CC_raw_\CC_impu_, and CC_impu_\CC_raw_).


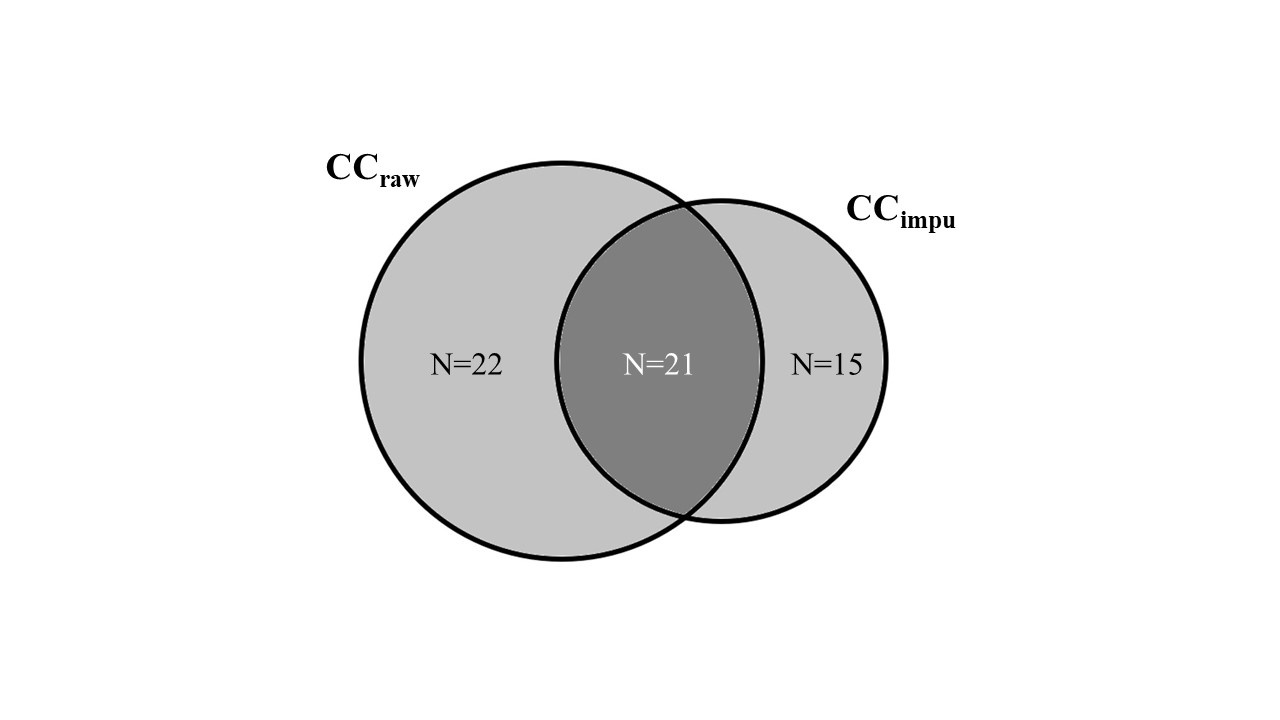

Supplement: Supplementary file 1 [file Data_Sheet_1.docx]
